# Evaluation of Acridine Orange Derivatives as DNA-Targeted Radiopharmaceuticals for Auger Therapy: Influence of the Radionuclide and Distance to DNA

**DOI:** 10.1038/srep42544

**Published:** 2017-02-13

**Authors:** Edgar Pereira, Letícia do Quental, Elisa Palma, Maria Cristina Oliveira, Filipa Mendes, Paula Raposinho, Isabel Correia, João Lavrado, Salvatore Di Maria, Ana Belchior, Pedro Vaz, Isabel Santos, António Paulo

**Affiliations:** 1 Centro de Ciências e Tecnologias Nucleares, Instituto Superior Técnico, Universidade de Lisboa, Estrada Nacional 10 (km 139,7), 2695-066 Bobadela LRS, Portugal; 2 Centro Química Estrutural, Instituto Superior Técnico, Universidade de Lisboa, Av. Rovisco Pais 1049-001 Lisboa, Portugal; 3 iMed.ULisboa, Faculdade de Farmácia, Universidade de Lisboa, Av. Prof. Gama Pinto, 1649-003 Lisboa, Portugal

## Abstract

A new family of ^99m^Tc(I)- tricarbonyl complexes and ^125^I-heteroaromatic compounds bearing an acridine orange (AO) DNA targeting unit was evaluated for Auger therapy. Characterization of the DNA interaction, performed with the non-radioactive Re and ^127^I congeners, confirmed that all compounds act as DNA intercalators. Both classes of compounds induce double strand breaks (DSB) in plasmid DNA but the extent of DNA damage is strongly dependent on the linker between the Auger emitter (^99m^Tc or ^125^I) and the AO moiety. The *in vitro* evaluation was complemented with molecular docking studies and Monte Carlo simulations of the energy deposited at the nanometric scale, which corroborated the experimental data. Two of the tested compounds, ^**125**^**I-C**_**5**_ and ^**99m**^**Tc-C**_**3**_, place the corresponding radionuclide at similar distances to DNA and produce comparable DSB yields in plasmid and cellular DNA. These results provide the first evidence that ^99m^Tc can induce DNA damage with similar efficiency to that of ^125^I, when both are positioned at comparable distances to the double helix. Furthermore, the high nuclear retention of ^**99m**^**Tc-C**_**3**_ in tumoral cells suggests that ^99m^Tc-labelled AO derivatives are more promising for the design of Auger-emitting radiopharmaceuticals than the ^125^I-labelled congeners.

In the latest decades, there has been progress in the overall control of cancer due to better screening and earlier detection, and as a result, patients are surviving longer. However, the incidence of some types of cancer is increasing, to the point that it is surpassing the progress achieved so far in the overall control of cancer. Target-specific and theranostic approaches to cancer, such as internal radionuclide therapy, can contribute to overcome some of these difficulties, offering new possibilities for more personalized treatment of malignant neoplastic diseases.

Radionuclide therapy is an anticancer modality based on the use of radiopharmaceuticals, which are drugs containing radionuclides emitting ionizing radiation (β^-^ and α particles or Auger-electrons)^1^,^2^,^3^. Many of these radionuclides are also gamma- or positron-emitters and thus, also useful for imaging via single-photon emission computerized tomography (SPECT) or positron emission tomography (PET), respectively. This possibility renders radionuclide therapy intrinsically suited to provide therapeutic effects and, simultaneously, to monitor non-invasively the therapeutic outcome in real-time. Therefore, radionuclide therapy has unique advantages within a theranostic approach for cancer when compared with other anticancer therapies.

In recent years, Auger-emitting radionuclides clinically used for SPECT imaging (*e.g.*^99m^Tc, ^123^I, ^67^Ga or ^111^In) have also been envisaged for selective and targeted radiotherapy^4^,^5^,^6^. The intrinsic advantages of Auger electrons when compared with other particulate radiation like β-particles, are related with their shorter penetration (<0.1 μm) in biological tissues, typically less than a cell diameter. Moreover, Auger electrons can induce biological effects that are characteristic of high-LET (Linear Energy Transfer) radiation, like α-particles, if the emitting radionuclide is located inside the cell nucleus. These biological effects are amplified by the emission of a cascade of Auger electrons per decay, producing clusters of DNA double strand breaks (DSBs) that can lead to severe DNA damage. These features make Auger-emitting radionuclides well-suited for the eradication of disseminated cancer metastases, with minimization of deleterious effect to non-target surrounding tissues.

A large variety of medically relevant Auger-emitting radionuclides is available^7^, spanning from *d*- or *f*- transition radiometals (*e.g*. ^67^Ga, ^89^Zr, ^90^Nb, ^99m^Tc, ^111^In, ^117m^Sn, ^119^Sb, ^195m^Pt, ^155^Tb, ^161^Tb) to radiohalogens (*e.g*. ^80m^Br, ^123^I, ^125^I), ensuring the exploration of a diversity of chemical structures to design multifunctional radioconjugates. From these Auger-emitters, ^111^In and ^125^I have been the most studied and several works have been published dealing with their potential usefulness for cancer therapy^4^,^5^,^6^,^8^,^9^. However, these results still have an ambiguous clinical translational potential in targeted radionuclide therapy.

The major challenge in the design of Auger-emitting radiopharmaceuticals is to obtain radioconjugates able to selectively transport a large number of radionuclide atoms to the nucleus of tumoral cells, ideally in close proximity to DNA. An attractive possibility to achieve this goal relies on radioconjugates carrying DNA intercalators that can deliver Auger electrons to short (sub)nanomolar distances to DNA inducing lethal DNA damage^10^,^11^,^12^,^13^,^14^,^15^. We and others have explored this approach for the Auger-emitting radionuclide ^99m^Tc^16^,^17^,^18^,^19^,^20^, which is still the most used radionuclide in diagnostic Nuclear Medicine. We have studied pyrazolyl-diamine ^99m^Tc (I) tricarbonyl complexes containing acridine orange (AO) intercalators and bombesin (BBN) analogues, which provided specificity towards the gastrin releasing peptide receptor (GRPR) overexpressed in prostate cancer cells^18^. One of these ^99m^Tc(I) complexes presented remarkably high cellular internalization and nuclear uptake and to the best of our knowledge, is the first and only example of a ^99m^Tc-bioconjugate that combines specific cell targeting with a pronounced nuclear internalization.

Following these encouraging results, we have carried out a more detailed study to evaluate the influence of the distance to DNA in the radiation-induced biological effects promoted by AO-containing ^99m^Tc(I) tricarbonyl complexes. We focused on pyrazolyl-diamine ^99m^Tc(I) tricarbonyl complexes containing alkyl linkers of different lengths between the AO unit and the bifunctional chelator.

As mentioned above, ^125^I is one of the most studied Auger-emitting radionuclides and preclinical studies have already proved its ability to exert *in vivo* antitumoral effects^9^. By contrast, preclinical reports for ^99m^Tc are scarce and present a narrower scope, and therefore additional studies are needed to have a clearer view of its potential in targeted radionuclide therapy. Taking this in consideration and for comparative purposes, this study was extended to ^125^I-labelled AO derivatives structurally related to the ^99m^Tc complexes (Fig. 1).

Here we present a detailed and multidisciplinary investigation of the radiation-induced effects of ^99m^Tc-complexes and structurally related ^125^I**-**labelled derivatives, which included spectroscopic characterization of DNA interaction, assessment of DNA damage *in vitro* and in living cells, cellular and nuclear internalization in tumoral cells and computational studies.

## Results

### Chemical and radiochemical synthesis

**Figure 2:**
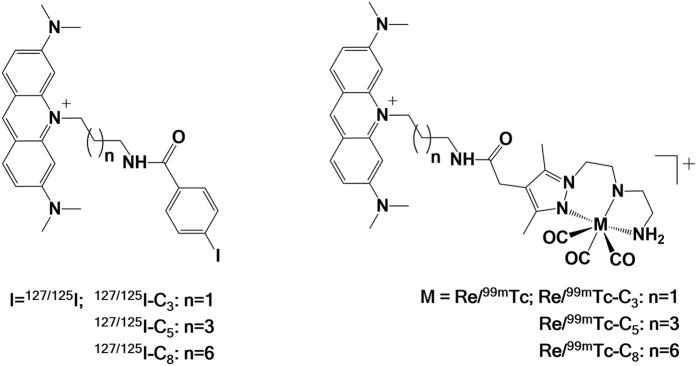
Chemical structures of the newly synthesized AO derivatives.

Structurally related ^99m^Tc- and ^125^I-labelled AO derivatives were designed sharing the same AO moiety and the same aminoalkyl spacers (Fig. 2). Thus, aminoalkyl-3,6-bis(dimethylamino)acridinium iodide precursors (**8–10**), containing three (C_3_), five (C_5_) and eight (C_8_) methylene units, were used as starting materials to obtain both classes of compounds (Supplementary Figs S1 and S3). These acridinium precursors were synthesized following previously described procedures^21^.

The iodinated AO derivatives, ^**127**^**I-C**_**3**_, ^**127**^**I-C**_**5**_ and ^**127**^**I-C**_**8,**_ were obtained by conjugation of the corresponding acridinium precursors (**8**–**10**) to tetrafluorophenyl-4-iodobenzoate (**11**). Their chemical characterization was done by common spectroscopic techniques (^1^H and ^13^C NMR) and ESI-MS. The radioactive congeners ^**125**^**I-C**_**3**_, ^**125**^**I-C**_**5**_ and ^**125**^**I-C**_**8**,_ were prepared by radioiododestannylation of the corresponding tributyltin precursors **Sn-C**_**3**_, **Sn-C**_**5**_ and **Sn**-**C**_**8**_ (Supplementary Fig. S1)^22^,^23^. After purification by RP-HPLC, these radiolabelled acridines were obtained with high specific activity and with a radiochemical purity exceeding 95% (see experimental details in Supplementary Information). The identity of the different radioiodinated AO derivatives was confirmed by HPLC co-elution with reference compounds ^**127**^**I-C**_**3**_, ^**127**^**I-C**_**5**_ and ^**127**^**I-C**_**8**_(Supplementary Fig. S2).

The synthesis of the ^99m^Tc(I) tricarbonyl complexes (^**99m**^**Tc-C**_**3**_, ^**99m**^**Tc-C**_**5**_ and ^**99m**^**Tc-C**_**8**_) was accomplished by a ligand-exchange reaction of *fac*-[^99m^Tc (H_2_O)_3_(CO)_3_]^+^ with the appropriate AO-containing pyrazolyl-diamine chelator (**L-C**_**3**_, **L-C**_**5**_ and **L-C**_**8**_) (Supplementary Fig. S3). The different chelators were synthesized based on methodologies previously described by our group, as detailed in Supplementary Information^21^,^24^. All ^99m^Tc-complexes were obtained with radiochemical purity higher than 99%, after RP-HPLC purification to remove the excess of the respective chelators.

The chemical identity of ^**99m**^**Tc-C**_**3**_, ^**99m**^**Tc-C**_**5**_ and ^**99m**^**Tc-C**_**8**_ was ascertained by comparison of their HPLC profiles with those of the corresponding rhenium complexes (**Re-C**_**3**_, **Re-C**_**5**_ and **Re-C**_**8**_) (Supplementary Fig. S4). This is a common practice in radiopharmaceutical chemistry, due to the chemical similarity between Tc and Re that form isostructural complexes with similar physicochemical properties. Re complexes were synthesized by reaction of the appropriate ligand with *fac*-[Re(H_2_O)_3_(CO)_3_]Br in a similar manner to the ^99m^Tc congeners. The rhenium complexes obtained were characterized by spectroscopic techniques (IR, ^1^H and ^13^C NMR) and ESI-MS.

### *In vitro* stability studies

RP-HPLC analysis showed that the radioiodinated AO derivatives were radiochemically stable up to 24 hours at 37 °C in 0.1 M Tris-HCl buffer (pH = 7.4) and up to 48 hours in in Dulbecco’s Modified Eagle Medium (DMEM), since no significant deiodination was detected. The AO-containing ^99m^Tc complexes also proved to be stable up to 24 hours in Tris-HCl buffer and up to 4 hours in DMEM (Supplementary Figs. S5 and S6). These results showed that the different radioactive compounds had the required *in vitro* stability under the conditions that were used for their biological evaluation as described below.

### Evaluation of DNA interaction by Spectroscopic Techniques

The interaction of the “cold” AO-containing compounds (iodinated derivatives and Re complexes) with calf thymus DNA (CT-DNA) was studied using UV-visible, fluorescence and circular dichroism (CD) spectroscopies, by monitoring the spectral changes in the presence of increasing amounts of DNA. The results for five compounds are presented in detail as Supplementary Information (Supplementary Table S4 and Supplementary Figs S7–S10). Because **Re-C**_**8**_ was obtained in very low yield, it was just used as a surrogate to identify the respective ^99m^Tc congener, and not for spectroscopic studies.

**Figure 3:**
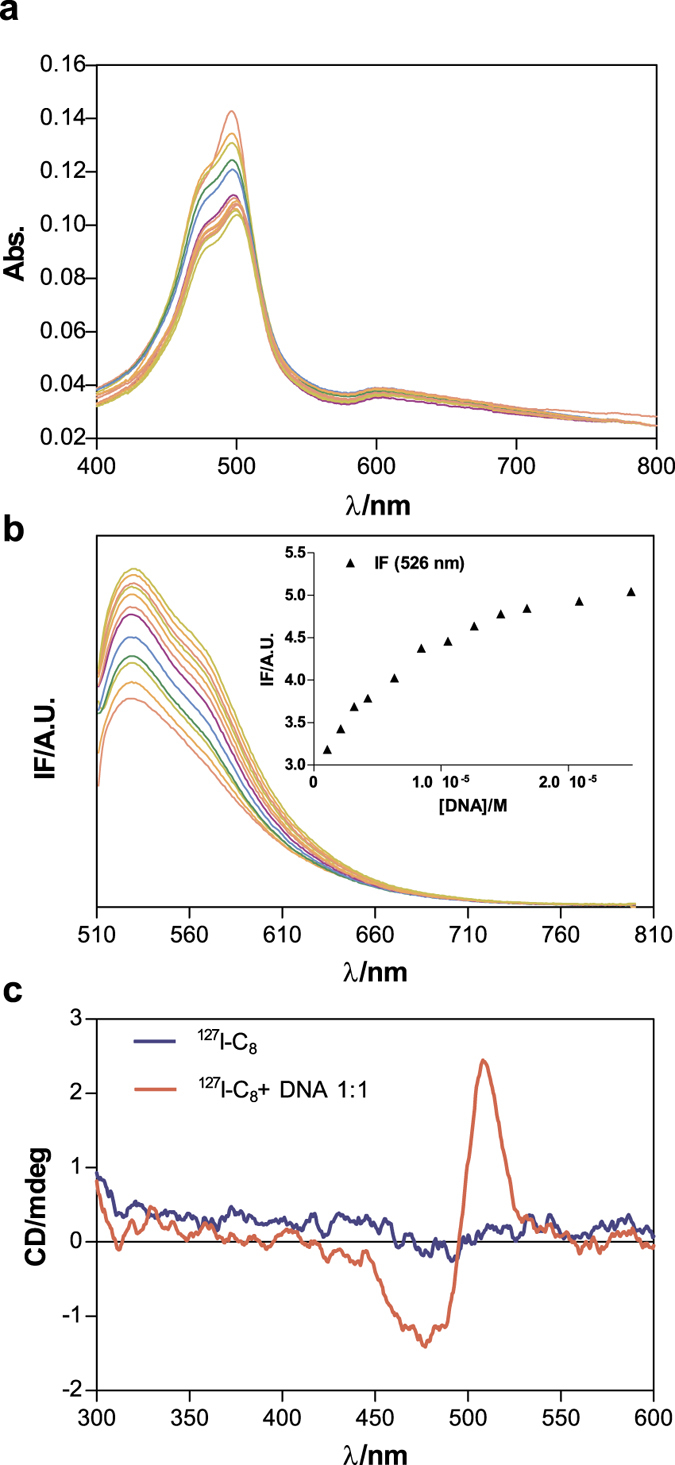
Spectroscopic evaluation of DNA interaction. (**a**) UV-Vis absorption spectra measured for solutions containing ligand**^127^I-C_8_**(ca. 6.0 μM) and increasing amounts of CT-DNA (ca. 3.1 mM). Inset: Variation of the absorption at 500 nm vs. [DNA]. (**b**) Fluorescence emission spectra measured for solutions containing **^127^I-C_8_** (ca. 5.5 μM) and increasing amounts of CT-DNA, after subtraction of blank emission spectra. Inset: Variation of the fluorescence intensity at 526 nm after correction for reabsorption and inner filter effects. Excitation at 505 nm; (**c**) Circular dichroism spectra measured for solutions containing**^127^I- C_8_**(ca. 25 μM) in the absence and presence of DNA (ratio DNA: **^127^I-C_8_ **= 1). Path length used was 2 cm.

As exemplified in Fig. 3 for ^**127**^**I-C**_**8**_, the most important spectroscopic fingerprints of the interaction of the compounds with CT-DNA are the following: i) bathochromic shifts in the UV-visible bands; ii) enhancement of the emission intensity in the fluorescence spectra; iii) induction of circular dichroism (CD) signal in the range where AO chromophores absorb. These spectroscopic changes are similar to the ones that we have previously reported for a Re complex related with **Re-C**_**3**_, **Re-C**_**5**_ and **Re-C**_**8.**_This complex presented a butylenic linker (C_4_) between the AO moiety and the pyrazolyl-diamine chelator and acted as a “perfect” DNA intercalator^17^. Taken together, data show that for all compounds the AO fragment acts as a DNA intercalator, independently of the spacer’s length and the presence of an iodinated benzoyl group or a pyrazolyl-diamine Re(I) tricarbonyl core. Unexpectedly, compounds ^127^**I-C**_**5**_ and **Re-C**_**5**_ showed CD bands between 400 and 550 nm, probably due to the folding of the spacer, which for both compounds is composed of five carbon atoms (see Supplementary Fig. S16).

All compounds showed fluorescence when excited at *ca.* 500 nm, which allowed titrations with increasing amounts of CT-DNA to quantify the binding affinity. In all cases, after addition of DNA a strong enhancement of the emission intensity was observed until saturation was reached, as exemplified for ^127^**I-C_8_** in Fig. 3b. The fluorescence titration data obtained for the other compounds are presented in Supplemental Information, together with the fitting of the data according to the Kaminoh^25^ and the McGhee von Hippel^26^ models (Supplementary Figs S11–S15). Based on these two models, the intrinsic binding constants (Table 1) were calculated as detailed in SI.

The fitting with the Kaminoh model always resulted in lower binding constants (range: 7.8–11.2 × 10^4^ M^−1^) than the McGhee von Hippel model (range: 35–60.2 × 10^4^ M^−1^). Nevertheless, it can be concluded that the Re complexes and iodinated derivatives display similar DNA binding ability when assessed using the same method. Moreover, these results show that all compounds have a moderate to high DNA binding affinity.

### Docking studies

Molecular modelling simulations were performed to acquire a comprehensive knowledge on the binding mode of the AO derivatives at an atomistic level, and to evaluate the effect of the side chain length on the distance between the DNA helical axis and the radionuclide. Based on the spectroscopic data, which revealed an intercalation binding mode both for the organometallic complexes and iodinated molecules, molecular docking of the AO derivatives was first performed on a small double-stranded DNA sequence, followed by molecular dynamics simulations. Thus, the starting model was the NMR solution structure of the d(ACGTACGT)_2_ sequence containing a bis-intercalating anthracycline (PDB code 1AL9)^27^, since it exhibits the required spacing between bases pairs to allow intercalation of the AO moiety.

**Figure 4:**
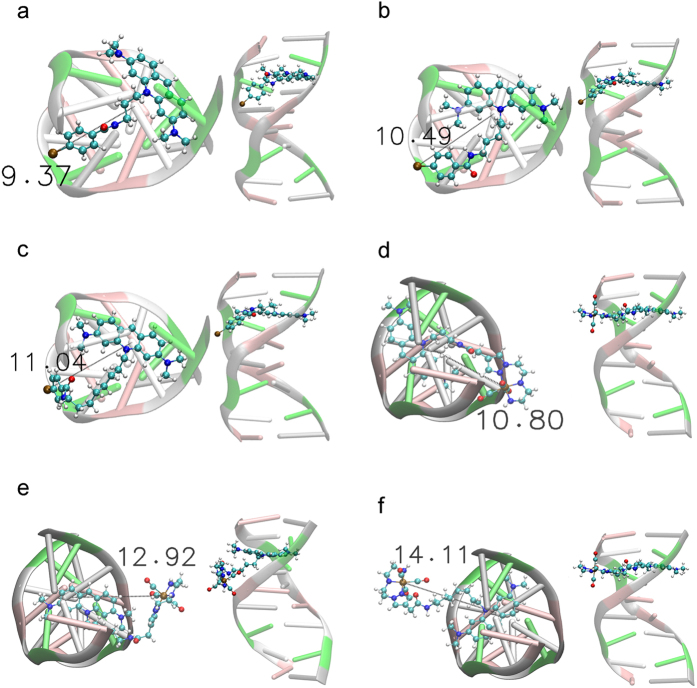
Top view and all structure view of the molecular docking top-ranked poses of the AO derivatives intercalated in the d(ACGTACGT)_2_ sequence. (**a**) ^125^**I-C_3_**; (**b**) ^125^I-C_5_; (**c**) ^125^I-C_8_; (**d**) ^99m^Tc-C_3_; (**e**) ^99m^Tc-C_5_ and (**f**) ^99m^Tc-C_8_. Distance between the **^125^I** or **^99m^Tc** atom relative to the DNA helical axis is displayed in Å.

The molecular docking studies were performed with Molecular Operating Environment (MOE)^28^ and validated by first docking the bis-intercalating anthracycline drug present in the NMR solution structure (Supplementary Fig. S17). Using the same protocol, ^**125**^**I-C**_**3**_, ^**125**^**I-C**_**5**_, ^**125**^**I-C**_**8**_, ^**99m**^**Tc-C**_**3**_, ^**99m**^**Tc-C**_**5**_ and ^**99m**^**Tc-C**_**8**_ were docked and the top-ranked positions are shown in Fig. 4.

The simulations corroborated that the AO derivatives intercalate between GC base pairs, stabilized by π-π stacking interactions with the nucleobases and by H-bonding between the intercalator side-chains and the phosphate backbone and/or nucleobases (Fig. 4). As shown in Table 2, the distances between the radionuclide atoms and the DNA helical axis are highly dependent on the side chain length. The calculated distances range between 9.37 and 11.04 Å for the iodinated molecules, and between 10.80 and 14.11 Å for the ^99m^Tc complexes.

Subsequently, to acquire a deeper knowledge of the binding of ligands and their dynamics, molecular dynamics (MD) simulations were run for the iodinated AO derivatives, using as starting point the molecular docking top-ranked pose, as reported in detail in the SI (Supplementary Figs S18–S20). Overall, the MD simulation results agreed with the docking studies, as the average distance between ^125^I and the ds-DNA helical axis is highly dependent of the length of the linker used to attach the AO unit, varying in the order C_3_ < C_5_ < C_8_ and ranging between 9.90 and 11.38 Å (Table 2).

In summary, the molecular modelling calculations showed that upon intercalation of the AO moiety, the radionuclide (^125^I or ^99m^Tc) is positioned at variable distances from the DNA helicoidal axis. For the same linker (C_3_, C_5_ or C_8_), the docking study showed that ^125^I is closer to the DNA axis than ^99m^Tc, which reflects the presence of a bulkier bifunctional chelator coordinated to the radiometal. Nevertheless, there is an overlap between the distance ranges determined for both radionuclides, allowing the desired comparison of the DNA damage induced by ^125^I *vs*^99m^Tc.

### DNA damage

#### Plasmid studies

Irradiation of plasmid DNA by the Auger-emitting radionuclides can cause single-strand (SSB), which transform supercoiled DNA (SC) into open circular DNA (OC) or double-strand breaks (DSB) which cause SC conversion into linear DNA (Lin).

The *in vitro* assessment of the presence and relative abundance of each DNA isoform by gel electrophoresis allows the quantification of SSBs and DSBs. DNA damage can be caused by direct interaction of DNA with the Auger electrons, but can also arise from indirect effects due to the formation of reactive oxygen species (ROS) generated by interaction of the Auger electrons with surrounding water molecules^29^,^30^. The role of direct versus indirect effects on the DNA damage can be estimated by performing the plasmid irradiation in the presence of radical scavengers, such as DMSO. These will react with the formed ROS and avoid the indirect DNA damage.

The ability of the radioiodinated derivatives (^**125**^**I-C**_**3**_, ^**125**^**I-C**_**5**_ and ^**125**^**I-C**_**8**_) and ^99m^Tc complexes (^**99m**^**Tc-C**_**3**_, ^**99m**^**Tc-C**_**5**_ and ^**99m**^**Tc-C**_**8**_) to induce DNA damage was studied by incubating the radiocompounds with supercoiled ϕX174 plasmid DNA at 4 °C. These experiments were also performed for Na^125^I and Na^99m^TcO_4_, which are simple chemical forms of each radionuclide unable to bind to DNA and which, therefore, should not promote extensive DNA damage. For a reliable comparison of the efficacy of each compound to induce DNA damage, plasmid DNA was exposed to similar numbers of increasingly accumulated decays. For this purpose, and due to the rather different ^99m^Tc and ^125^I half-lives, two different approaches were used. For the shortest half-lived ^99m^Tc (T_1/2_ = 6.02 h) the same concentration of ϕX174 plasmid was exposed for 24 h to increasing amounts of each complex (5 to 500 μCi). After incubation, the samples were analysed by gel electrophoresis and the different DNA isoforms quantified by densitometry. By contrast, ϕX174 plasmid DNA was exposed to a fixed initial amount (~40 μCi) of each ^125^I-labelled compound up to 28 days, and aliquots of the reaction mixture were analysed every 7 days.

**Figure 5:**
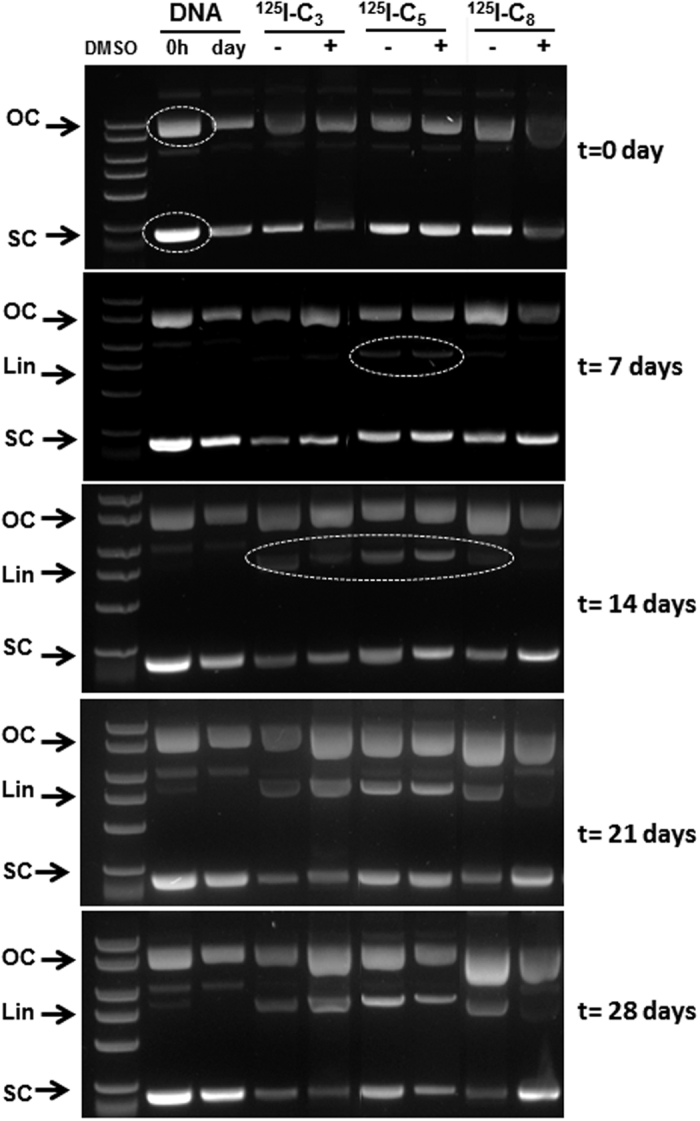
Cleavage of supercoiled ϕX174 DNA by ^125^I-C_3_, ^125^I-C_5_ and ^125^I-C_8_. Incubation was performed for 28 days at 4 °C in Tris-HCl buffer (pH 7.4) in the presence or absence of DMSO (0.2 M). SC, OC and Lin are supercoiled, open circular and linear forms of DNA, respectively. “DNA 0 h” is the DNA control without any incubation and “DNA day” is the DNA control without compounds but incubated at 4 °C in Tris-HCl buffer (pH 7.4) for the same time period as the ^125^I compounds.

**Figure 6:**
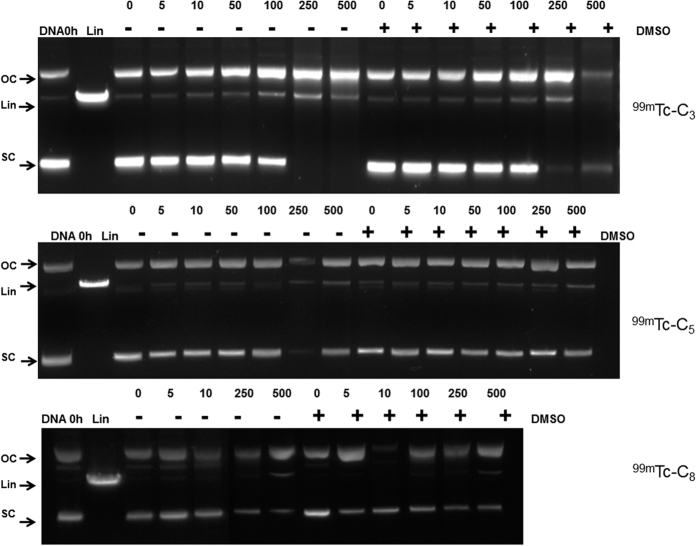
Cleavage of supercoiled ϕX174 DNA by ^99m^Tc-C_3_, ^99m^Tc -C_5_ and ^99m^Tc -C_8_. Incubation was performed for 24 hours at 4 °C in Tris-HCl buffer (pH 7.4) with different activities of the complexes (in μCi), in the presence or absence of DMSO (0.2 M). SC, OC and Lin are supercoiled, open circular and linear forms of DNA, respectively. “DNA 0 h” is the DNA control without any incubation.

Representative examples of gel electrophoresis images obtained for ϕX174 plasmid DNA, after exposure to the different compounds, are presented in Figs 5 and 6 and Supplementary Figs S21 and S22. Na^125^I and Na^99m^TcO_4_ even at the maximum radioactivity values used for the other tested compounds, did not induce detectable alterations in the relative proportions of the different DNA isoforms when compared with the control sample (no radioactive compound) (Supplementary Figs S21 and S22). Hence, these compounds do not induce significant DNA damage.

In the absence of DMSO, exposure of plasmid DNA to the radioiodinated derivatives, ^**125**^**I-C**_**3**_, ^**125**^**I-C**_**5**_ and ^**125**^**I-C**_**8**_, led to the appearance of the Lin isoform (Fig. 5 and Supplementary Fig. S23). While DMSO did not prevent the formation of the Lin isoform for ^**125**^**I-C**_**3**_ and ^**125**^**I-C**_**5,**_ it blocked the formation of this isoform upon plasmid irradiation with ^**125**^**I-C**_**8**_.

^**99m**^**Tc-C**_**3**_ led to a more significant appearance of linear DNA and augmented proportion of the OC isoform when compared with the other ^99m^Tc(I) tricarbonyl complexes, as seen in Fig. 6 and Supplementary Fig. S24. For ^**99m**^**Tc-C**_**3**,_ activities higher than 200 μCi led to disappearance of all SC DNA and conversion into OC and Lin isoforms, showing the high ability of this complex to induce DNA damage. In the case of ^**99m**^**Tc-C**_**5**_, the Lin isoform was also formed, but without the complete conversion of the SC-DNA, even at the highest activities tested. ^**99m**^**Tc-C**_**8**_ demonstrated a weaker ability to induce alterations in the DNA isoforms, since only at 500 μCi the Lin conformation was observed.

For a quantitative view of the DNA damage induced by each compound, the number of DSBs per plasmid molecule as a function of the accumulated decays/mL was calculated, based on the conversion of supercoiled plasmid DNA into linear DNA, as detailed in the experimental section (Supplementary Fig. S25)^30^. In the absence of DMSO, ^**125**^**I-C**_**3**_, ^**125**^**I-C**_**5**_, ^**125**^**I-C**_**8**_, and ^**99m**^**Tc-C**_**3**_ led to well-defined linear plots, reflecting the formation of a reasonably high number of DSBs that steadily increased with the rise of accumulated decays. By contrast, ^**99m**^**Tc-C**_**5**_ and ^**99m**^**Tc-C**_**8**_ provoked less DNA damage and lower DSB values were obtained. From the plots two radiosensitivity parameters were calculated: the D_0_ values, i.e. the number of decays required to produce one DSB, and the yield of DSBs per decay (Y(DSB)) (see details in the SI). These values are summarized in Table 3.

For the radioiodinated compounds, there was a clear influence of the ^125^I-distance to DNA in the DSBs yield, either in the absence of presence of DMSO, which decreases monotonically with increasing distance. For ^**125**^**I-C**_**3**_ and ^**125**^**I-C**_**5**_, the Y(DSB) values were rather similar in the presence or absence of DMSO (^**125**^**I-C**_**3**,_ 0.073 *vs* 0.079; ^**125**^**I-C**_**5**_, 0.048 *vs* 0.037) demonstrating that the DNA damage induced by these compounds is mainly due to direct effects. Moreover, for these compounds the percentage of the OC isoform remained fairly constant along the different time points (Supplementary Fig. S23). This indicates that the steadily increasing formation of linear DNA is due to DSBs occurring directly in SC DNA and caused by the Auger electrons emitted by the intercalated ^**125**^**I-C**_**3**_ and ^**125**^**I-C**_**5**_. By contrast, the ROS scavenger DMSO completely blocked the DNA damage induced by ^**125**^**I-C**_**8**_, with the DSB yield decreasing from 0.033 to a virtually null value. In addition, in the presence of DMSO, ^**125**^**I-C**_**8**_ led to a remarkable increase of the OC isoform, which apparently follows the rate of formation of the linear isoform. This behaviour corroborates that the DNA damage being caused by ^**125**^**I-C**_**8**_involves mainly indirect effects.

Our results show that there is a sharply defined threshold distance dictating the prevalence of direct DNA damage for this family of ^125^I-labelled DNA intercalators, as relatively close values of 10.49 and 11.04 Å for ^**125**^**I-C**_**5**_ and ^**125**^**I-C**_**8**_, respectively, were found for the ^125^I- DNA axis distance by the docking simulations. The same trend was described by Balagurumorthy *et al*., who have verified that ^125^I-labelled Hoechst derivatives acting as minor groove binders produce DSBs exclusively by indirect effects when the ^125^I atom is located at a distance >12 Å from the DNA, and DNA damage by direct effects at shorter distances^31^.

The DSB yield in the absence of DMSO for ^**99m**^**Tc-C**_**3**_ was 0.0336 per decay while for ^**99m**^**Tc-C**_**5**_ and ^**99m**^**Tc-C**_**8**_ the values were almost one order of magnitude lower and span between 0.0022 to 0.0067 DSBs per decay (Table 3). Upon addition of DMSO, the DSB yields by ^**99m**^**Tc-C**_**3**_ dropped from 0.0336 to 0.0224 per decay, showing that ^**99m**^**Tc-C**_**3**_ produces a considerable amount of DSBs by direct effects although there is also a small contribution of indirect effects (Table 3 and Supplementary Fig. S25). For ^**99m**^**Tc-C**_**5**_ and ^**99m**^**Tc-C**_**8**_, the presence of DMSO also led to a decrease of the DSB yields that become almost negligible (0.0022 and 0.0034, respectively), indicating a much lower ability to produce DSBs by direct effects than ^**99m**^**Tc-C**_**3**_. These data confirm that the mean distance of the ^99m^Tc atom to the DNA axis has a strong influence in the nature and extension of the DNA damage, namely in production of DSBs.

The results obtained for ^**99m**^**Tc-C**_**3**_ are in agreement with those recently reported by Freudenberg *et al*. for ^99m^Tc-HYNIC-DAPI that induces DSBs through direct interaction with DNA with very similar DSB yield (0.03 per decay of ^99m^Tc)^32^,^33^. These similarities indicate that the ^99m^Tc atom must be placed by both compounds at comparable mean distance from the DNA axis.

#### Nanodosimetric studies

Meaningful insights about the effectiveness of the biological effects induced by therapeutic radionuclides can be obtained by modelling and simulation using Monte Carlo (MC) methods of the DNA geometry, the structure of the ionizations produced along the particles´ track and the energy deposited by the emitted particles in biological media (cells and DNA). During the last decade, the state-of-the-art has rapidly evolved towards the development of sophisticated computational tools simulating the physical, chemical and biological stages of the effects induced by ionizing radiation. At the same time, the need to develop new dosimetric quantities (beyond the absorbed dose) at the nanoscopic scale was pinpointed^34^.

In order to rationalize the results of the plasmid DNA experiments, MC simulations were performed to calculate the deposited energy in nanometric DNA volumes. The goal was to compare qualitatively the calculated deposited energy with the DSB yields measured in the plasmid DNA experiments (assuming that for a given radiation quality the locally deposited energy would be proportional to the ionization cluster size). Deposited energies were calculated in a volume corresponding to a DNA segment of 10 base pairs of length embedded in its nucleosome, both modelled as liquid water cylinders with nanometric dimensions^35^,^36^. The source term was simulated as an anisotropically emitting Auger electrons source. The Auger electrons were emitted at different distances to the DNA axis (see Supplementary Fig. S26), using the ^125^I or ^99m^Tc Auger, Coster-Kronig and super Coster-Kronig energy spectra^37^. In the MC simulations performed only the physical stage was taken into account^38^, as detailed in the experimental section, and therefore the role of indirect effects on DNA damage was not estimated.

**Figure 7:**
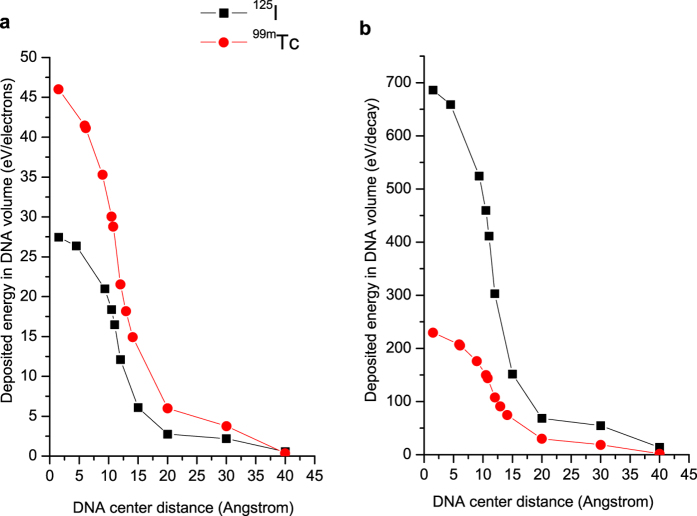
Deposited energies in the DNA segment with the decay sources (^125^I and ^99m^Tc) at different distances from the DNA center: (**a**) deposited energies per Auger electron emitted; (**b**) deposited energies normalized per decay.

Figure 7 shows the deposited energy in the DNA segment for ^125^I and ^99m^Tc as a function of the distance of each radioactive atom to the DNA axis. The deposited energy was calculated per emitted electron and per decay (5 and 25 Auger electrons emitted for ^99m^Tc and ^125^I, respectively). The deposited energy per emitted electron is always greater for ^99m^Tc than ^125^I, particularly for the shortest distances to DNA (Fig. 7a). For example, the deposited energy calculated for the ^125^I atom at a distance of about 11 Å from the center of the DNA segment is comparable with the one induced by ^99m^Tc at a distance of about 13 Å. This means that the Auger electrons emitted by ^99m^Tc can be more effective in producing direct DSBs when compared with those emitted by ^125^I. The normalization of the MC results to the real number of Auger electrons emitted per decay shows that ^125^I leads to higher absorbed energies than ^99m^Tc at similar distances to the DNA (Fig. 7b). However, these values become more comparable for the range of distances (roughly 10–15 Å) calculated by the molecular docking simulations for the ^125^I- and ^99m^Tc-labelled AO derivatives described herein.

The analysis of Fig. 7b confirms that there is a steep variation of the deposited energy as a function of the DNA distance for both radionuclides, suggesting the existence of a critical distance, from which the direct effects stop being effective. Based on the experimental DSB yields obtained for ^**125**^**I-C**_**3**_, ^**125**^**I-C**_**5**_ and ^**125**^**I-C**_**8**_ in the presence and absence of DMSO, it is quite evident that at distances to DNA of approximately 11.0 Å (^**125**^**I-C**_**8**_) DSBs are mostly generated by indirect effects, while at 10.5 Å (^**125**^**I-C**_**5**_) the production of DSBs occurs essentially by direct effects. These results point out that the critical distance should be in the range 10.49–11.04 Å, which is roughly in agreement with the 12 Å value proposed by Balagurumorthy *et al*.^31^. For the ^99m^Tc(I) complexes, the assignment of such critical distance based on the experimental results is not so straightforward. First, the complex ^**99m**^**Tc-C**_**3**_, characterized by the shortest ^99m^Tc to DNA distance (10.80 Å), produced a reasonably high number of DSBs due to a combination of direct and indirect effects, although with a prominent influence of direct effects. Second, the DSB yields induced by ^**99m**^**Tc-C**_**5**_ and ^**99m**^**Tc-C**_**8**_were much lower, even in the absence of DMSO, with a consequently higher uncertainty in its estimation and an increased difficulty in the quantitative assessment of the DMSO influence.

**Figure 8:**
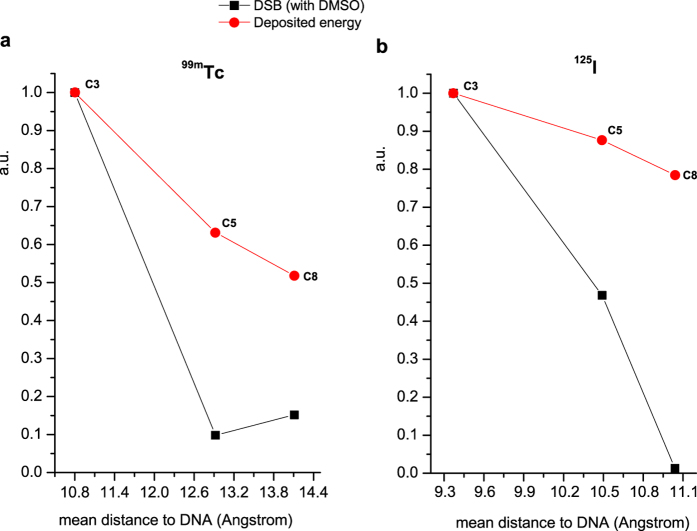
Correlation between the experimental DSB yield per decay and the calculated deposited energy: (**a**) ^99m^Tc(I) complexes and (**b**) ^125^I-labelled derivatives. All values are normalized to their maximum values.

The experimental DSB yields by direct effects found for ^**125**^**I-C**_**8**_, ^**99m**^**Tc-C**_**5**_ and ^**99m**^**Tc-C**_**8**_ are very close to zero, being clearly inferior to the values expected based on the MC simulations, as shown in Fig. 8 that represents the variation of DSB yield and the calculated deposited energy versus the distance to DNA. This indicates that the theoretical calculations overestimate the direct DNA damage, as previously observed by other authors for ^125^I-labelled DNA groove binders^31^,^39^. These discrepancies can eventually result from the use of a rigid rod-like model for DNA in the MC calculations, while the supercoiled ϕX174 plasmid DNA has a more flexible and dynamic structure.

#### Cell studies

As discussed above, compounds^**125**^**I-C**_**5**_ and ^**99m**^**Tc-C**_**3**_ position the respective radionuclides at similar distances from DNA and induce relatively comparable DSBs yields in supercoiled plasmid DNA. These results prompted us to evaluate both compounds in tumoral cell lines aiming to investigate their ability to induce DNA DSBs under more “realistic” biological conditions where different barriers need to be crossed to reach the target DNA, enclosed in the nucleus and embedded in different proteins.

### Cellular and nuclear uptake

**Figure 9:**
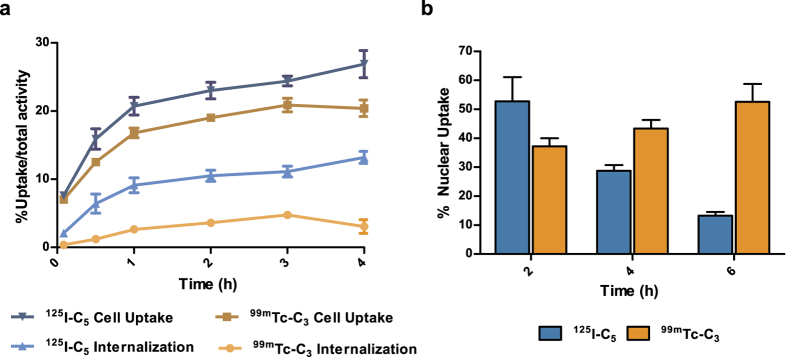
Cellular and nuclear uptake of**^125^I-C_5_**and **^99m^Tc-C3** in PC3 cells: (**a**) Cellular uptake and internalization. The total activity associated with the cell (cellular uptake) as well as the intracellular activity (internalized) expressed as a percentage of total activity applied. (**b**) Nuclear uptake expressed as a percentage of cell-associated activity.

First, the subcellular localization of ^**125**^**I-C**_**5**_ and ^**99m**^**Tc-C**_**3**_ was evaluated by quantitative gamma-counting measurements. The results were obtained by incubation of the two radiocompounds with PC3 cells for up to 4 h and are presented in Fig. 9.

Both compounds (^**125**^**I-C**_**5**_ and ^**99m**^**Tc-C**_**3**_) exhibited high time-dependent uptake in PC3 cells (Fig. 9a). For ^**125**^**I-C**_**5**_, the maximum value of cell uptake was 27% with a maximum internalization of 13%; for ^**99m**^**Tc-C**_**3**_, up to 20% of the total applied activity was cell-associated but only 4.8% was internalized after 3 h of incubation. These results show that ^**125**^**I-C**_**5**_ internalizes in a higher extent than ^**99m**^**Tc-C**_**3**_, with 45 and 23% of the cellular uptake corresponding to internalized compound, respectively.

The nuclear uptake and retention of ^**125**^**I-C**_**5**_ and ^**99m**^**Tc-C**_**3**_ was evaluated and exhibited quite different patterns. As shown in Fig. 9b, the nuclear internalization of ^**99m**^**Tc-C**_**3**_ steadily increased over time, from 37 to 52% between 2 and 6 h of incubation. By contrast, ^**125**^**I-C**_**5**_ showed a fast and high nuclear internalization, as roughly 50% of the activity associated with the cell is in the nucleus after 2 h of incubation. Nevertheless, the nuclear fraction decreased from 53 to 13% after 6 h of incubation, although the cellular internalization remained almost constant along the 2–6 h incubation period (Fig. 9a). These results indicate that there is a significant release of the ^125^I-activity from the nucleus, in opposition to the ^99m^Tc-activity that is retained after the entrance of ^**99m**^**Tc-C**_**3**_into the nucleus. Interestingly, Zalutsky *et al*. have reported the same type of behaviour for ^125^I-labelled protein-based compounds^40^ *versus* related ^111^In-labelled compounds^41^. As invoked by these authors, this difference could be explained by a higher stability of metallic complexes against nuclear degradation and/or their higher ability to be trapped in the cell nuclei, when compared with iodinated compounds.

### Cellular DNA damage

After evaluation of their internalization in human tumoral cells, ^**99m**^**Tc-C**_**3**_ and ^**125**^**I-C**_**5**_were assessed for their ability to induce DSBs *in vivo* in the same cell line. Cells were incubated with the compounds for 2 and 24 h periods and the formation of γ-H2AX foci was analyzed by immunofluorescence. The formation of these foci is an early event that follows the induction of DSBs, and results from the phosphorylation of the X isoform of histone H2A at serine-139 by phosphoinositide 3-kinases^42^,^43^.

**Figure 10:**
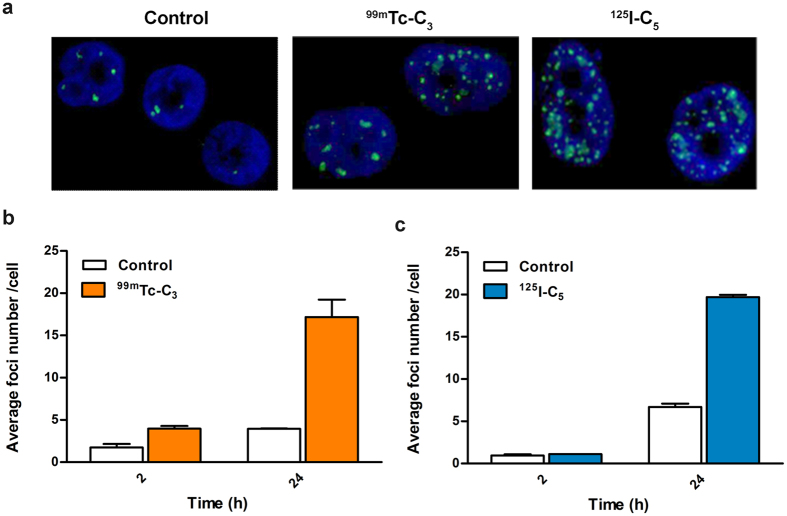
Induction of DNA damage by ^99m^Tc-C_3_ and ^125^I-C_5_ in PC3 cells: (**a**) Fluorescence images of untreated PC3 cells (control) or cells exposed to ^99m^Tc-C_3_ and ^125^I-C_5_ for 24 h. Cells were immunostained for γ-H2AX. DAPI was used to visualize the nuclei; (**b**) and (**c**) Average number of foci per cell in PC3 cells incubated during 2 and 24 hours with ^99m^Tc-C_3_ and ^125^I-C_5_, respectively.

For each incubation time, the average number of foci was calculated from the distribution of foci number per cell (see Supplementary Fig. S27). As can be seen in Fig. 10, the exposure of cells to ^**99m**^**Tc-C**_**3**_ and ^**125**^**I-C**_**5**_ resulted in a significant increase (p < 0.05) in the average number of γ-H2AX foci compared with untreated control cells, particularly after 24 h of incubation. The number of γ-H2AX foci after exposure to an internalized radionuclide depends on the balance between the repaired lesions and the DSBs that are continuously formed as the radionuclide decays^44^. The influence of DNA repair is expected to be more important for longer incubation times, as the discontinuous irradiation of cells with γ-radiation leads to a maximum number of γ-H2AX foci at around 30 min, which then diminishes due to repair of the lesions^4^. For ^**99m**^**Tc-C**_**3**_ and ^**125**^**I-C**_**5**_ the amount of foci largely increased from 2 to 24 h of incubation, suggesting that the DNA damage induced by both compounds exceeded the cellular capacity for its repair. ^**125**^**I-C**_**5**_ induced the formation of a higher number of foci per cell when compared with ^**99m**^**Tc-C**_**3**_, in line with the highest DSB yield that was found for ^**125**^**I-C**_**5**_in the plasmid DNA experiments. However, it is important to emphasize that a reliable quantitative interpretation of these data would require microdosimetric calculations, which are out of the scope of the present work.

## Discussion

Herein, we have described an unprecedented comparative *in vitro/in silico* study of structurally related compounds carrying the Auger emitters ^125^I and ^99m^Tc, aiming to assess the influence of the distance to DNA and nature of the radionuclide on the DNA damage. For this purpose, we have focused on AO derivatives that were successfully labelled with the desired radionuclide using a 4-[^125^I]iodobenzoyl group or a pyrazolyl-diamine ^99m^Tc(I) tricarbonyl moiety, linked to the intercalating AO unit through alkyl linkers of different length. Based on the ^127^I and Re congeners, we have proved that all compounds act as DNA intercalators and have similar high binding affinity, independently of the linker and group used to incorporate the radioactive label (^125^I or ^99m^Tc). This was a crucial issue for the feasibility of the intended comparative study, as it assured that all the tested radioactive compounds were virtually quantitatively bound to DNA, taking into consideration their specific activity. This way, the probability of DNA damage due to free molecules randomly distributed in solution could be minimized.

Both classes of compounds, ^99m^Tc- and ^125^I-labelled AO derivatives, are able to induce DSBs in plasmid DNA but the damage extent and the role of direct effects are strongly dependent on the linker used to attach the Auger emitting radionuclide to the AO moiety. In general, the calculated DSB yields decreased monotonically with the distance to DNA; being however quite similar for ^**99m**^**Tc-C**_**5**_ and ^**99m**^**Tc-C**_**8**_, although these complexes position the ^99m^Tc atom at different distances from DNA (12.92 and 14.11 Å, respectively) according to the molecular docking studies. Probably, this result reflects the low number of DSBs detected at these rather large distances, which contributed to the increase in uncertainty in the calculation of the respective DSB yields. Overall, the docking and MC simulations corroborated the experimental data, despite some tendency of the theoretical simulations to overestimate the DNA damage at longer DNA-radionuclide distances.

For the ^125^I-labelled AO derivatives, we could demonstrate that there is a “cut-off” value for the distance at which the DSBs switch from being exclusively due to direct effects (shortest distances) to being exclusively produced by indirect effects (longest distances). Such distance lies between 10.49 and 11.04 Å, corresponding to the ^125^I-distances to DNA calculated for ^**125**^**I-C**_**5**_ and ^**125**^**I-C**_**8,**_respectively. For the ^99m^Tc complexes, the existence of a similar “cut-off” distance was not evident, as the formation of DSBs already involves the contribution of indirect effects in the case of ^**99m**^**Tc-C**_**3**_, which places the ^99m^Tc atom at the shortest distance to DNA and induces the highest DSB yield by direct effects. The reason behind these differences could not be clarified by the MC simulations, as it did not consider the involvement of indirect effects and also used exclusively the Auger spectra of each radionuclide in the calculations (without any contribution of the respective γ-spectra).

The docking simulations revealed that the compounds ^**125**^**I-C**_**5**_ and ^**99m**^**Tc-C**_**3**_ place the corresponding radionuclide at relatively similar distances from the DNA (10.49 and 10.80 Å, respectively), which rendered these two compounds particularly interesting for our comparative study. In fact, these two ^99m^Tc and ^125^I-labelled AO derivatives showed a rather similar ability to induce DSBs in plasmid DNA by direct effects. Moreover, this ability was translated into living cells where the ^99m^Tc complex showed an enhanced retention when compared with the congener ^125^I-labelled AO derivative.

Based on related chemical structures, our results demonstrated for the first time that ^99m^Tc can induce DNA damage with an efficiency that parallels that of ^125^I, when positioned at similar distances from the DNA. These results clearly indicate that the DNA damage induced by ^**99m**^**Tc-C**_**3**_ involves direct effects by Auger electrons, due to its emission in close proximity to the target DNA. These unprecedented results point out that ^99m^Tc has the necessary requisites to be explored as a theranostic radionuclide and encourage further investigation in Auger therapy.

Furthermore, the retention of ^**99m**^**Tc-C**_**3**_ in the nucleus of tumoral cells was higher than that of ^**125**^**I-C**_**5**_ suggesting that ^99m^Tc-labelled AO derivatives are more promising than their ^125^I-labelled counterparts in the design of Auger-emitting radiopharmaceuticals. ^**99m**^**Tc-C**_**3**_ is not expected to display by itself preferential tumor cell uptake relatively to normal cells. However, as we have demonstrated elsewhere, this class of complexes can be endowed with specificity towards tumoral cells upon functionalization with targeting biomolecules, while retaining the ability to be internalized by the nucleus. Altogether, these breakthroughs give impetus to further investigation of ^99m^Tc-labelled AO derivatives as multifunctional radiopharmaceuticals for anticancer Auger therapy.

## Methods

### Chemical and Radiochemical Synthesis

Details of chemical/radiochemical synthesis, purification and characterization of the different compounds are provided in Supplementary Information.

### Spectroscopic evaluation of DNA binding ability

UV-Visible absorption (UV-Vis) spectra were recorded on a Perkin-Elmer Lambda 35 spectrophotometer at room temperature. Fluorescence spectra were measured on Horiba Jobin Yvon fluorescence spectrometer model FL 1065 at room temperature. The circular dichroism (CD) spectra were recorded at 25 °C on a Jasco J-720 spectropolarimeter with UV-Vis (200–700 nm) photomultipliers (EXEL-308). Millipore water was used for the preparation of solutions and TRIS-HCl buffer (0.1 M, pH = 7.4) was used in all experiments. Methanol (or acetonitrile:methanol 2:1 for complex **Re-C**_**5**_) from Panreac was used for the preparation of the compounds stock solutions, which were always used within a few hours. The concentration of DNA in base pairs was determined by UV−Vis absorbance using the molar absorption coefficient at 260 nm (6600 M^−1^cm^−1^). The CT-DNA sodium salt was purchased from Sigma and used as received. The stock solutions (~1 mg/mL) were prepared by dissolution in TRIS buffer. The solutions were used within 3 days after their preparation. The amount of organic solvent was kept below 1% (v/v, fluorescence) or 5% (v/v, CD) in each experiment.

### Docking studies

#### Model generation

The NMR solution structure of the d(ACGTACGT)_2_ sequence, containing a bis-intercalating anthracycline (PDB code 1AL9)^27^ was chosen as the starting model for the molecular modelling simulation. The structure was prepared with the Molecular Operating Environment (MOE) v.2013.08 software package^28^ by removing waters, ligand and adding the missing hydrogen atoms and protonation states with Protonate 3D application tool, at 300 K, pH 7 and salt concentration of 0.1 M at the GB/VI electrostatic formalism. The acridine ligands were constructed in MOE and optimized by Density Functional Theory (DFT) with B3LYP parameterization of the density functional and the 6–31 + G(d, p) basis set in addition to the external DGDZVP basis set for iodine atoms, using the Gaussian 03 software package^45^. Restrained Electrostatic Potential (RESP) charge derivation of the acridine ligands was obtained with the Antechamber application present in AmberTools15 software package^46^. Ligand topology was obtained from the Automated Topology Builder (ATB), version 2.0^47^,^48^. Partial charges used in MD simulations were obtained by substituting the charges assigned by the ATB topology with the RESP charges^47^. The remaining parameters for the ligand and DNA were obtained from the generalized AMBER03 force field^49^,^50^.

#### Molecular docking

The docking studies were performed in MOE using Induced Fit docking protocol, Alpha Triangle placement and London dG scoring function. Final poses were subjected to a final refinement in the receptor pocket with the AMBER99 force field, at an RMS gradient threshold of 0.01 and rescoring with London dG free-energy scoring function to retain 100 ranked poses. All ds-DNA structure was used as potential docking site.

#### Molecular dynamics simulations

The models obtained from the molecular docking simulations containing the acridine ligands were subjected to MD simulations using the GROMACS simulation package 4.5.5 and applying the AMBER03 force field^49^,^50^. The ds-DNA:ligand structures were inserted in a cubic box, with at least 10 Å between the ds-DNA and the simulation box edge. The system was solvated with TIP3PBOX water model^51^ and neutralized by adding the required K^+^ ions. After energy minimization of the MD box with the steepest descent method, a 100 ps *NVT* equilibration run followed at 298 K (spatially restraining all oligonucleotide atoms) was performed. The equilibration stage involved a 1 ns run and sampling the *NPT* ensemble (T = 298 K, p = 1 bar) and, finally the unconstrained MD simulation of the system was performed for 50 ns in the same isothermic-isobaric ensemble. In all MD runs, the particle-mesh-Ewald (PME) formalism was applied to the long-range electrostatic interactions. The short-range electrostatic cut-off was 12 Å, and the same length was applied for the van der Waals (vdW) interactions. All bonds were constrained with the Lincs algorithm. Nosé-Hoover and Parrinello–Rahman constraints were applied to control the temperature and isotropic pressure (τ_T_ = 0.2, τ_p_ = 5.0 ps, and β = 4.5 × 10^−5^ bar^−1^). Energy and pressure corrections for the vdW cutoff were also applied. Visualizations and images were obtained in MOE v.2013.08 and/or with VMD (Visual Molecular Dynamics) software, v.1.9^52^.

### Plasmid studies

#### DNA damage evaluation

The plasmid DNA used for gel electrophoresis experiments was ϕX174 (Promega). Linear DNA was obtained by digestion with the single-cutter restriction enzyme *Xho*I and used as reference in agarose gel electrophoresis. DNA cleavage activity was evaluated by monitoring the conversion of supercoiled plasmid DNA (SC – form I) to open circular DNA (OC– form II) and linear DNA (Lin –form III). The experimental setup was different for the two types of radiolabelled compounds.

For the ^99m^Tc complexes, ^**99m**^**Tc-C_3_**, ^**99m**^**Tc-C_5_** and ^**99m**^**Tc-C_8_**, due to the short semi disintegration period, aliquots of DNA were incubated with increasing amounts of radiolabelled complexes. Each reaction mixture was prepared by adding 2 μL (200 ng) of supercoiled DNA and radiolabelled complex (activities ranging from 5 to 500 μCi) in Tris-HCl 0.1 M (pH 7.4) to a final volume of 30 μL. When appropriate, DMSO (0.2 M) was added to the reaction mixture. Samples were typically incubated at 4 °C, for 24 h. After incubation, 3 μL of DNA loading buffer (0.25% bromophenol blue, 0.25% xylene cyanol, 30% glycerol in water, Applichem) were added to each tube and the sample was loaded onto a 0.8% agarose gel in TBE buffer (89 mM Tris–borate, 1 mM EDTA pH 8.3) containing Gel Red (0.5 mg.mL^−1^) (Biotium, Hayward, CA, USA). Controls of non-incubated and of linearized plasmid were loaded on each gel electrophoresis. The electrophoresis was carried out for c.a. 2 h at 100 V.

For the radioiodinated compounds, ^**125**^**I-C**_**3**_, ^**125**^**I-C**_**5**_ and ^**125**^**I-C**_**8,**_ profiting from the longer semi disintegration period, DNA was incubated with the radiolabelled compounds, and aliquots were removed and analysed at different time points. The incubation was performed with 2 μg of supercoiled DNA and the desired compound (ca. 40 μCi) in Tris-HCl 0.1 M (pH 7, 4) in a final volume of 100 μL. When appropriate, DMSO (0.2 M) was added to the reaction mixture. This mixture was incubated at 4 °C, for 28 days, and at every 7 days an aliquot of 8 μL was removed. To this aliquot, DNA loading buffer was added and the samples were loaded onto agarose-gel and electrophoresis was performed as described above.

Bands were visualised under UV light and images captured using an AlphaImagerEP (Alpha Innotech). Peak areas were measured by densitometry using AlphaView Sofware (Alpha Innotech). The photos chosen for this publication were rearranged to show only the relevant samples. All samples in each figure were obtained from the same run. Peak areas were used to calculate the percentage (%) of each isoform (SC, OC and Lin), with a correction factor for the SC form to account for its lower staining. Those percentages were used to estimate the number of SSBs and DSBs, as detailed in the supplementary information.

### Nanodosimetric studies by Monte Carlo simulations

Electrons fluxes and deposited energy were calculated through the MCNP6 MC simulations^53^. Considering the electron transport, the ENDF/B VI.8 database contains cross sections for atomic excitation, electron elastic scattering, subshell electro-ionization and *bremsstrahlung*, and is able to simulate electron energies down to 10 eV^54^. An important development in the MCNP6 MC Code version is the introduction of a single-event electron transport for energies below 1 keV, in a completely different approach than the one used for higher energies with the condensed-history method^54^, making it a more suitable MC code for nano dosimetric calculations.

MC simulations were undertaken to calculate the deposited energy in nanometric DNA volumes (see Supplementary Information for details on the adopted geometrical setup). In these types of simulations only the physical stage (space distribution of ionization excitations and elastic scattering between the first 10^−15^ s and 10^−13^ s of interaction) was taken into account. Pre-chemical and chemical stages (diffusion and interaction of water radicals and molecular products) were not considered^37^, and for this reason the indirect effects on DSBs were not estimated through MC simulations.

### Cellular studies

PC3 human prostate cancer cells (ECACC, England, UK) were grown in DMEM containing GlutaMax supplemented with 10% heat-inactivated fetal bovine serum and 1% penicillin/streptomycin antibiotic solution (all from Gibco-Invitrogen), in a humidified atmosphere of 95% air and 5% CO_2_ at 37 °C (Heraeus, Germany).

#### Internalization and cellular uptake

PC3 cells were seeded at a density of 0.2 million per well in 24 well-plates and allowed to attach overnight. The cells were incubated at 37 °C for a period of 5 min to 4 h with about 0.2 μCi of the radiocompound in 0.5 mL of assay medium (MEM with 25 mM N-(2-hydroxyethyl)piperazine-N-ethanesulfonic acid (HEPES) and 0.2% BSA). Incubation was terminated by washing the cells with ice-cold assay medium. Cell surface-bound radiocompound was removed by two steps of acid wash (50 mM glycine, HCl/100 mM NaCl, pH 2.8) at room temperature for 4 min. The pH was neutralized with cold PBS with 0.2% BSA, and subsequently the cells were lysed with 1 M NaOH for 10 min at 37 °C to determine internalized radiocompound.

#### Nuclear Uptake

PC3 cells were seeded at a density of 1 million per well in 6 well-plates and allowed to attach overnight. The cells were incubated at 37 °C for a period of 2, 4 and 6 h with about 2 μCi of the radiocompound in 2 mL of assay medium (MEM with 25 mM HEPES and 0.2% BSA). At each time interval, cells in radioactive media were removed from the plates by scrapping and collected into a 2 mL tube. The unbound radioactive compound was removed by centrifugation of the cell suspension at 2100 g for 3 min at 4 °C, followed by washing the cellular pellet with ice-cold PBS with 0.2% BSA. The activity of cellular pellet was measured, using a gamma counter, to quantify the total cellular uptake of the radiocompound. The pellet was then ressuspended in 2 mL of ice‐cold cell lysis buffer (10 mM Tris, 1.5 mM MgCl_2_, 140 mM NaCl) containing 0.1% of IGEPAL-ca 630 (Sigma) and incubated on ice for 10 min to disrupt the cell membrane. After the lysis, the suspension was centrifuged at 1300 g for 2 min at 4 °C, the supernatant (cytoplasm) was separated from the pellet (nuclei) and the activity in both fractions measured. The nuclear uptake was expressed in percentage of total cellular uptake.

#### γ-H2AX assay and foci analysis

PC3 cells were seeded at a density of 10000 cells per well in an eight-well chamber slide and allowed to attach overnight. Cells were incubated with ^**99m**^**Tc-C**_**3**_(50 μCi) and ^**125**^**I-C**_**5**_ (8 μCi) for 2 and 24 h at 37 °C. PC3 cells were washed three times with PBS, and fixed with 4% formaldehyde in PBS for 15 min. After washing with PBS, cells were permeabilized with Triton X-100 (0.5%) at room temperature for 5 min followed by two washing steps with 1% BSA in PBS. Then cells were incubated with an anti-γ-H2AX primary antibody (mouse anti-γ-H2AX (ser139), Stressgen, bioreagents Corp., Canada) at 2 μg/mL for 1 h. After being washed twice with 1% BSA in PBS, cells were incubated with a FITC-conjugated anti-mouse secondary antibody (Santa Cruz Biotechnology, USA) at 1 mg/ml for 1 h, followed by three washing steps with PBS. After incubation with Hoechst (Sigma-Aldrich, St. Louis, USA) at 1 μg/ml for 5 min, cells were finally mounted in anti-fade mounting media (Vectashield H-100, Vector Laboratories, Burlingame, Canada).

Cells were analysed under 64x magnification in a Zeiss Axioplan2 imaging microscope. Several images were randomly collected in each slide. Image analysis of γ-H2AX foci was performed using the freeware Cellprofiler^55^. At least 200 nuclei were analyzed per experiment per dose. Statistical analysis was performed with Origin 7.5 software. For each experiment, the two population means were compared using the non-parametric Mann-Whitney test.

## Additional Information

**How to cite this article:** Pereira, E. *et al*. Evaluation of Acridine Orange Derivatives as DNA-Targeted Radiopharmaceuticals for Auger Therapy: Influence of the Radionuclide and Distance to DNA. *Sci. Rep.* **7**, 42544; doi: 10.1038/srep42544 (2017).

**Publisher's note:** Springer Nature remains neutral with regard to jurisdictional claims in published maps and institutional affiliations.

## Supplementary Material

Supplementary Information

## Figures and Tables

**Figure 1:**
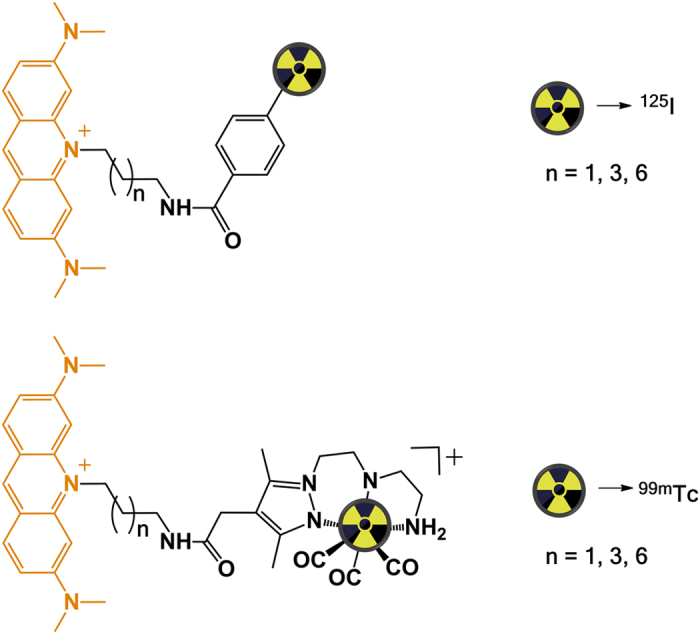
Schematic drawing of the ^125^I- and ^99m^Tc-labelled AO derivatives studied. AO is represented in orange.

**Table 1:** Intrinsic binding constants (K/M^−1^) and other parameters obtained from the application of the Kaminoh^25^ and McGhee von Hippel^26^ models to the fluorescence emission data.

	^127^I-C_3_	^127^I-C_5_	^127^I-C_8_	Re-C_3_	Re-C_5_
Kaminoh					
K × 10^4^	7.8 ± 2.1	9.5 ± 2.8	11.2 ± 1.4	11.2 ± 0.3	9.21 ± 1.5
*I*_*sat*_ × 10^7^	6.7 ± 0.3	6.12 ± 0.06	5.86 ± 0.1	4.54	7.33
*R*^*2*^	0.995	0.983	0.996	0.990	0.989
McGhee von Hippel					
K × 10^4^	38 ± 6	47 ± 9	35 ± 3	59.3 ± 0.1	60.2 ± 0.2
n	1.3 ± 0.2	2.4 ± 0.3	1.25 ± 0.08	3.7 ± 0.6	2.48 ± 0.03
R^2^ (nr points)	0.871 (4)	0.864 (4)	0.838 (10)	0.769 (5)	0.997 (5)

**Table 2:** Distance between the radionuclide in the intercalated AO derivatives and the DNA helical axis obtained by molecular modelling simulations.

Linker	Distance/Å		
	^125^I(a)	^99m^Tc(a)	^125^I(b)
C_3_	9.37	10.80	9.90
C_5_	10.49	12.92	11.06
C_8_	11.04	14.11	11.38

^(a)^Molecular docking-top ranked pose.

^(b)^Average distance in last 30 ns of the molecular dynamics simulation.

**Table 3:** Radiosensitivity parameters (D_0_ and DSB yield) of supercoiled ϕX174 DNA upon exposure to the^125^I derivatives and ^99m^Tc(I) complexes, in the presence or absence of DMSO.

Compound	Mean distance to DNA (Å)	D_0_ (x10^13^ Decays/mL)		DSB yield/decay (10^−2^)	
		−DMSO	+DMSO	−DMSO	+DMSO
^125^I-C_3_	9.37	4.57 ± 0.41	4.28 ± 0.77	7.30 ± 0.65	7.90 ± 1.42
^125^I-C_5_	10.49	6.94 ± 0.20	8.96 ± 0.18	4.80 ± 0.14	3.70 ± 0.07
^125^I-C_8_	11.04	10.30 ± 1.23	234.00 ± 192.00	3.30 ± 0.39	0.10 ± 0.08
^99m^Tc-C_3_	10.80	3.33 ± 0.50	5.00 ± 0.45	3.36 ± 0.50	2.24 ± 0.20
^99m^Tc-C_5_	12.92	33.30 ± 6.32	50.00 ± 3.00	0.34 ± 0.06	0.22 ± 0.01
^99m^Tc-C_8_	14.11	16.70 ± 1.50	33.30 ± 2.00	0.67 ± 0.06	0.34 ± 0.02
