# Supplementary material for: Evaluation of Acridine Orange Derivatives as DNA-Targeted Radiopharmaceuticals for Auger Therapy: Influence of the Radionuclide and Distance to DNA
Source: Sci Rep. 2017 Feb 13;7:42544. doi: 10.1038/srep42544 (PMC5304164; doi:10.1038/srep42544)
Supplement: Supplementary Information [file srep42544-s1.pdf]

Supplementary information

Evaluation of Acridine Orange Derivatives as DNA-Targeted Radiopharmaceuticals for Auger Therapy: Influence of the Radionuclide and Distance to DNA

Edgar Pereira[1¥], Letícia do Quental[1¥], Elisa Palma[1,2], Maria Cristina Oliveira[1], Filipa Mendes[1], Paula Raposinho[1], Isabel Correia[2], João Lavrado[3], Salvatore Di Maria[1], Ana Belchior[1], Pedro Vaz[1], Isabel Santos[1], António Paulo[1*]

[1]Centro de Ciências e Tecnologias Nucleares, Instituto Superior Técnico, Universidade de Lisboa, Estrada Nacional 10 (km 139,7), 2695-066 Bobadela LRS, Portugal

[2]Centro Química Estrutural, Instituto Superior Técnico, Universidade de Lisboa, Av. Rovisco Pais 1049-001 Lisboa, Portugal

[3]iMed.ULisboa, Faculdade de Farmácia, Universidade de Lisboa, Av. Prof. Gama Pinto, 1649-003 Lisboa

[¥] These authors contributed equally to this work

**Corresponding author:* apaulo@ctn.tecnico.ulisboa.pt

Table of contents

1. Chemical and radiochemical synthesis
    - 1.1 Reagents and general procedures
    - 1.2 Chemistry and radiochemistry
        - 1.2.1 Synthesis of iodinated/radioiodinated AO derivatives
        - 1.2.2 Synthesis of AO-containing Re and ^{99m}Tc complexes
    - 1.3 *In vitro* stability studies
2. Spectroscopic evaluation of the DNA binding ability
    - 2.1 UV-Vis absorption spectra
    - 2.2 Fluorescence titrations
    - 2.3 Circular dichroism spectra
3. Molecular modelling
    - 3.1 Molecular docking validation
    - 3.2 Molecular Dynamics simulations
4. DNA damage
    - 4.1 Estimation of DSBs
    - 4.2 DNA electrophoresis
5. Monte Carlo simulations
6. Cell Studies
7. References

1. Chemical and Radiochemical Synthesis

1.1. Reagents and general procedures

All reagents were analytical grade and were used without further purification. Unless stated otherwise, the syntheses of the ligands and complexes were carried under nitrogen atmosphere, using standard *Schlenk* techniques and dried solvents. HPLC-grade solvents were used for HPLC purification and analysis.

tert-Butyl N-(2-((*tert*-butoxy)carbonyl)(2-(4-(2-(2,5-dioxopyrrolidin-1-yl)-2-oxoethyl)-3,5-dimethylpyrazol-1-yl)ethyl)amino)ethyl) carbamate (**12**)[1] and *fac*-$[Re(CO)_3(H_2O)_3]Br$ were synthesized as described elsewhere[2].

Sodium [^{125}I]iodide was obtained from Perkin Elmer, USA, as a non-carrier added solution in 0.1 M aqueous NaOH with radionuclidic purity > 99% and specific activity of 643.8 GBq/mg. Na[$^{99m}TcO_4$] was eluted from a commercial ^{99}Mo/^{99m}Tc generator (Drytec, GE Healthcare) using a 0.9% saline solution.

^{1}H and ^{13}C NMR spectra were recorded on a Varian Unity 300 MHz spectrometer, using $CDCl_3$ or CD_3OD as solvents. ^{1}H and ^{13}C chemical shifts are given in ppm and were referenced to the residual solvent resonances relative to $SiMe_4$. The spectra were assigned with the help of 2D NMR spectroscopy (H–H COSY, and 1H–13CHSQC).

IR spectra were recorded in the range 4000–400 cm^{-1} as KBr or CsI pellets on a Bruker Tensor 27 spectrometer.

Electrospray ionisation mass spectrometry (ESI-MS) was performed using a Bruker HCT electrospray ionization quadrupole ion trap mass spectrometer.

Elemental analyses were performed on a Perkin-Elmer automatic analyzer.

Analytical thin layer chromatography (TLC) was carried out on silica gel 60 F254 (Merck) or Aluminum oxide 0.2mm (Macherey-Nagel) plates and spots were visualized under UV light (254 nm).

Column chromatography was performed with silica gel 60 (Merck) or aluminum oxide 90 neutral (Macherey-Nagel). Solid-phase extraction (SPE) columns (Waters Sep-Pak C18 cartridges) were obtained from Waters Corp (Milford, MA, USA)

Reverse phase high performance liquid chromatography was carried out on a PerkinElmer system equipped with a biocompatible quaternary pump (series 200), an UV/vis detector (SPD-10 AV, Shimadzu, UV detection at 254 nm) and a radioactivity detector (LB 509 or LB-507A, Berthold).

The purification and analytical control of radioiodinated compounds was accomplished using an EC-Nucleosil C18 column (250x4 mm, 10 µm, Macherey Nagel) eluted at a flow rate of 1.0 mL/min with an isocratic mixture of 0.1% TFA (v/v) in water and 0.1% TFA (v/v) in acetonitrile (40:60) for 35 min (method 1).

Analytical control of ligands (**L-C$_3$, L-C$_5$, L-C$_8$**) and Re/^{99m}Tc complexes, and purification of ^{99m}Tc complexes was achieved in an analytical EC-Nucleosil C18 column (250x4 mm, 5 µm, Macherey Nagel) with a flow rate of 0.5 mL/min. The solvents were 0.1% TFA (v/v) in water as eluent A and methanol as eluent B.

The gradient used for analysis was: 0–3 min, 100% A; 3–3.1 min, 100%–75% A; 3.1–9 min, 75% A; 9–9.1 min 75%–66% A; 9.1–18 min, 66%–0% A; 18–25 min, 0% A; 25–25.1 min, 0%–100% A; 25.1–30 min, 100% A (method 2).

The gradient used for purification was*:* 0–3 min, 75% A; 3–3.1 min, 75%– 66% A; 3.1–23 min, 66%–10% A; 23–28min, 10% A; 28–28.1 min, 10%–0% A; 28.1–33 min 0% A; 33 –33.1 min, 0%–75% A; 33.1–38 min, 75% A (method 3).

1.2. Chemistry and radiochemistry

1.2.1. Synthesis of iodinated/radioiodinated AO derivatives

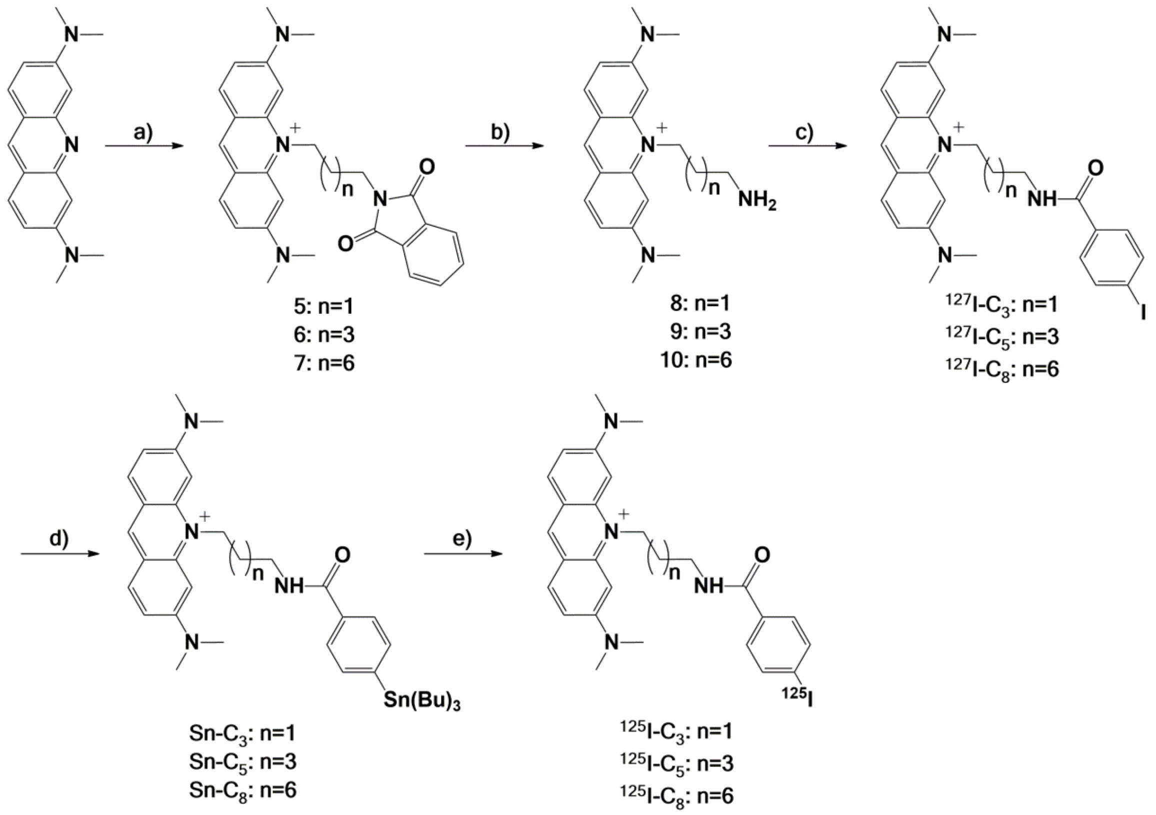

Figure S1. Synthesis of iodinated/radioiodinated AO derivatives.

i) **2, 3** or **4** , *p*-xylene, N_2, reflux, 24h
ii) 1. $NH_2NH_2.H_2O$, dry MeOH, N_2, reflux, 5 days. 2. HCl (37%), NaOH (4M)
iii) 1. DMF, DIPEA, 0ºC, 30 min. 2. Tetrafluorophenyl-4-iodobenzoate (**11**), r.t., overnight
iv) $PdCl_3(PPh_3)_2$, $(Sn(Bu)_3)_2$, dry DMF, N_2, reflux, overnight
v) $[^{125}I]NaI$, H_2O_2 (3% w/v), HCl (1M), MeOH, r.t., 10 min

^{127}I heteroaromatic compounds bearing an AO intercalating moiety

N-(3-iodopropyl) phthalimide ***(2)****, 6-bis(dimethylamino)-10-(3-(1,3-dioxoisoindolin-2-yl)propyl)acridinium iodide* ***(5)*** *and 10-(3-aminopropyl)-3,6-bis(dimethylamino)acridinium iodide* ***(8)*** were synthesized as previously reported[3].

N-(5-iodopentyl) phthalimide ***(3)*** Potassium iodide (2.17 g, 13.07 mmol) was added to a solution of *N*-(5-bromopentyl) phthalimide (2.02 g, 6.82 mmol) in dry acetone (40 ml) and the mixture was heated at reflux for 48 hours under nitrogen atmosphere. The formed precipitate was discarded by filtration and the remaining filtrate was evaporated and dried under reduced pressure, to give ***3*** as a yellow oil (320 mg, 6.77 mmol, 99%). ^{1}H NMR (300 MHz, $CDCl_3$, Me_4Si) δ 1.44 (m, 2H, C*H*$_2$), 1.69 (m, 2H, C*H*$_2$), 1.88 (m, 2H, C*H*$_2$), 3.18 (t, 2H, CH_2), 3.68 (t, 2H, C*H*$_2$), 7.71 (t, 2H, C*H*), 7.84 (m, 2H, C*H*).

N-(8-iodooctyl) phthalimide ***(4)***. Potassium iodide (457.0 mg, 2.75 mmol) was added to a solution of N-(8-bromooctyl) phthalimide (549.0 mg, 1.62 mmol) in dry acetone (12 ml) and the mixture was heated at reflux for 24 hours under nitrogen atmosphere. The formed precipitated was discarded by filtration and the remaining filtrate was evaporated and dried under reduced pressure, to give **4** as a yellow oil (516.0 mg, 1.33 mmol, 82%). ^{1}H NMR (300 MHz, $CDCl_3$, Me_4Si) δ 1.29 (m, 8H, C*H*$_2$), 1.65 (m, 2H, C*H*$_2$), 1.78 (m, 2H, C*H*$_2$), 3.13 (t, 2H, C*H*$_2$), 3.63 (t, 2H, C*H*$_2$), 7.69 (t, 2H, C*H*), 7.80 (m, 2H, C*H*).

3,6-bis(dimethylamino)-10-(5-(1,3-dioxoisoindolin-2-yl) pentyl)acridinium iodide ***(6)***. To a solution of *N-(5-iodopentyl) phthalimide* ***(3)*** (665.0 mg, 1.94 mmol) in *p*-xylene (15 ml), acridine orange (**1**, 280.0 mg, 1.05 mmol) was slowly added. The resulting mixture was heated at reflux for 72 hours under nitrogen atmosphere. After cooling, the formed red precipitate was filtered and dried under reduced pressure to give ***6*** as a dark-red solid (577.0 mg, 0.95 mmol, 90%). ^{1}H NMR (300 MHz, $CDCl_3$, Me_4Si) δ 1.81 (m, 4H, C*H*$_2$), 2.04 (m, 2H, C*H*$_2$), 3.31 (s, 12H, C*H*$_3$), 3.71 (t, 2H, C*H*$_2$), 4.78 (t, 2H, C*H*$_2$), 6.57 (s, 2H, C*H*), 7.01 (d, 2H, C*H*), 7.67-7.87 (m, 6H, C*H*) 8.61 (s, 1H, C*H*).

3,6-bis(dimethylamino)-10-(8-(1,3-dioxoisoindolin-2-yl)octyl)acridinium iodide ***(7)***. To a solution of *N-(8-iodooctyl) phthalimide* ***(4)*** (516.1 mg, 1.33 mmol) in *p*-xylene (10

ml) acridine orange (176.5 mg, 0.67 mmol) was slowly added. The resulting mixture was heated at reflux for 72 hours under nitrogen atmosphere. After cooling, the red precipitate formed was filtered and dried under reduced pressure to give **7** as a dark-red solid (215.0 mg, 0.33 mmol, 49%). ^{1}H NMR (300 MHz, $CDCl_3$, Me_4Si) δ 1.36 (m, 8H, CH_2), 1.62 (m, 2H, CH_2), 1.94 (m, 2H, CH_2), 3.31 (s, 12H, CH_3), 3.64 (t, 2H, CH_2), 4.77 (t, 2H, CH_2), 6.56 (s, 2H, C*H*), 7.03 (d, 2H, C*H*), 7.71 (d, 2H, C*H*), 7.82 (d, 2H, C*H*), 7.89 (d, 2H, C*H*), 8.71 (s, 1H, C*H*).

10-(5-aminopentyl)-3,6-bis(dimethylamino)acridinium iodide ***(9).*** Hydrazine monohydrate (2.3 ml, 47.42 mmol) was added to a suspension of *3,6-bis(dimethylamino)-10-(5-(1,3-dioxoisoindolin-2-yl) pentyl)acridinium iodide* (**6**) (2.02 g, 3.35 mmol) in dry methanol (135 ml). The mixture was heated at reflux for 5 days under nitrogen atmosphere. After cooling, the mixture was acidified with 25 ml of concentrated HCl (37%) resulting in the formation of a precipitate of phtalic acid hydrazide that was discarded by filtration. The pH of the filtrate was adjusted to 14 by addition of a 4 M NaOH solution. The solvent was evaporated and the compound extracted with CH_2Cl_2 (4x5 mL) and the combined organic phases were dried over Na_2SO_4 and evaporated. The residue was taken up in CH_2Cl_2 (5 ml) and diethyl ether (35 ml) and centrifuged for 10 minutes at 2.500 rpm. After cooling at 0°C for 18 hours the supernatant was discarded and the sediment resuspended in cold methanol. After evaporation of methanol, **9** was obtained as a red solid (594.0 mg, 1.24 mmol, 37%) ^{1}H NMR (300 MHz, CD_3OD, Me_4Si) δ 1.66 (s, 4H, CH_2), 1.93 (m, 2H, CH_2), 2.72 (t, 2H, CH_2), 3.28 (s, 12H, CH_3), 4.55 (m, 2H, CH_2), 6.51 (m, 2H, C*H*), 7.15 (m, 2H, C*H*), 7.79 (m, 2H, C*H*), 8.54 (m, 1H, C*H*). ^{13}C NMR (300 MHz, CD_3OD, Me_4Si) δ 25.31, 26.70, 33.06, 40.93, 42.27, 48.55, 93.48, 115.38, 118.34, 134.21, 143.80, 144.01, 157.15. ESI-MS *m/z* calcd for $C_{13}H_{14}NO_2I$ ($[M]^+$): 351.3, found 351.8.

10-(8-aminooctyl)-3,6-bis(dimethylamino)acridinium iodide ***(10)***. Hydrazine monohydrate (1.0 ml, 20.62 mmol) was added to a suspension of *3,6-bis(dimethylamino)-10-(8-(1,3-dioxoisoindolin-2-yl) octyl)acridinium iodide* (**7**) (1.0 g, 1.55 mmol) in dry methanol (65 ml). The mixture was heated at reflux for 5 days under nitrogen atmosphere. After cooling, the mixture was acidified with 12 ml of concentrated HCl (37%) resulting in the formation of a precipitate of phtalic acid hydrazide that was discarded by filtration. The pH of the filtrate was adjusted to 14 by

addition of a 4 M NaOH solution. The solvent was evaporated and the compound was extracted with CH_2Cl_2 (4x5 mL) and the combined organic phases were dried over Na_2SO_4 and evaporated. The residue was taken up in CH_2Cl_2 (5 ml) and diethyl ether (35 ml) and centrifuged for 10 minutes at 2.500 rpm. After cooling at 0ºC for 18 hours the supernatant was discarded and the sediment resuspended in cold methanol. After evaporation of methanol **10** was obtained as a red solid (444.0 mg, 0.85 mmol, 55%).^{1}H NMR (300 MHz, CD_3OD, Me_4Si) δ 1.37 (m, 4H, C*H$_2$*), 1.49 (m, 4H, C*H$_2$*), 1.61 (m, 2H, C*H$_2$*), 1.89 (m, 2CH, C*H$_2$*), 2.67 (t, 2H, C*H$_2$*), 3.25 (s, 12H, C*H$_3$*), 4.54 (t, 2H, C*H$_2$*), 6.49 (s, 2H, C*H*), 7.15 (d, 2H, C*H*), 7.78 (d, 2H, C*H*), 8.51 (s, 1H, C*H*). ^{13}C NMR (300 MHz, CD_3OD, Me_4Si) δ 26.88, 27.82, 27.91, 30.35, 30.49, 32.43, 40.92, 42.08, 49.85, 93.53, 115.42, 118.36, 134.23, 143.83, 144.02, 157.16. ESI-MS *m/z* calcd for $C_{16}H_{20}NO_2I$ ($[M]^+$): 393.3, found 393.5.

Tetrafluorophenyl-4-iodobenzoate ***(11)*** was synthesised according to a reported procedure with minor modifications [4]. Briefly, to a solution of tetrafluorophenol (**TFP**, 401.8 mg, 2.42 mmol) in 1,4-dioxane (10 ml) were added 4-iodobenzoic acid (500.0 mg, 2.02 mmol), and EDC (463.9 mg, 2.42 mmol). The mixture was kept stirring for 24 hours and the activated ester was extracted with CH_2Cl_2. The organic phase was dried over $MgSO_4$, filtered and the solvent was evaporated. The dry residue was purified by silica gel column chromatography (ethyl acetate:petroleum ether; 1:6). Tetrafluorophenyl-4-iodobenzoate (**5**) was obtained as a colourless crystalline solid after evaporation of solvent from the collected fractions under reduced pressure (737.0 mg, 1.86 mmol, 92%).^{1}H NMR (300 MHz, $CDCl_3$, Me_4Si) δ 7.05(m, 1H, C*H*), 7.92(m, 4H, C*H*).

10-(3-(4-iodobenzamide)propyl))-3,6-bis(dimethylamino) acridinium iodide (**^{127}I-C$_3$**). DIPEA (2 eq, 320 µl, 1.80 mmol) was added to a solution of **8** (0.41 g, 0.90 mmol) in DMF (9 mL) and the solution was kept stirring for 30 min at 0ºC. Thereafter the reaction mixture was warmed to room temperature and tetrafluorophenyl 4-iodobenzoate (1.5 eq, 0.54 g, 1,37 mmol) was added and the mixture was kept stirring overnight. Solvent was removed under reduced pressure and the dry residue purified by column chromatography on aluminium oxide (MeOH:CH_2Cl_2; 1:9) to give **^{127}I-C$_3$** as a red solid (0.32 g, 0.65 mmol, 72%). ^{1}H NMR (300 MHz, $CDCl_3$, Me_4Si) δ 2.34 (m, 2H, C*H$_2$*), 3.18 (s, 12H, C*H$_3$*) 3.89 (m, 2H, C*H$_2$*), 4.96 (t, 2H, C*H$_2$*), 6.67 (s, 2H, C*H*), 7.01

(d, 2H, C*H*), 7.75 (d, 2H, C*H*), 7.83 (d, 2H, C*H*), 8.15 (d, 2H, C*H*), 8.41 (s, 1H, C*H*). ^{13}C NMR (300 MHz, $CDCl_3$, Me_4Si) δ 26.07, 37.45, 41.06, 47.17, 93.07, 98.77, 114.27, 117.14, 129.96, 133.01, 133.35, 137.73, 142.51, 142.89, 155.79, 167.18. ESI-MS *m/z* calcd for $C_{27}H_{30}$ N_4OI_2 ([M]+): 553.1, found 553.2.

10-(5-(4-iodobenzamide)pentyl))-3,6-bis(dimethylamino) acridinium iodide (**^{127}I-C_5**) DIPEA (2 eq, 90 µl, 0.50 mmol) was added to a solution of **9** (0.12 g, 0.25 mmol) *in* DMF (12 mL) and the solution was kept stirring for 30 min at 0ºC. Thereafter the reaction mixture was warmed to room temperature and tetrafluorophenyl 4-iodobenzoate (1.5 eq, 0.15 g, 0.38 mmol) was added and the mixture was kept stirring overnight. Solvent was removed under reduced pressure and the dry residue purified by column chromatography on aluminium oxide ($MeOH:CH_2Cl_2$; 1:9) to give **^{127}I-C_5** as a red solid (0.10 g, 0.18 mmol, 72%). ^{1}H NMR (300 MHz, $CDCl_3$, Me_4Si) δ 1.85 (m, 2H, C*H₂*), 2.00 (m, 4H, C*H₂*), 3.29 (s, 12H, C*H₃*), 3.60 (t, 2H, C*H₂*), 4.78 (t, 2H, C*H₂*), 6.63 (s, 2H, C*H*), 7.04 (d, 2H, C*H*), 7.76-7.80 (m, 4H, C*H*), 7.95 (d, 2H, C*H*), 8.20 (t, 1H, N*H*), 8.48 (s, 1H, C*H*). ^{13}C NMR (300 MHz, $CDCl_3$, Me_4Si) δ 24.44, 25.77, 28.85, 39.35, 41.36, 48.39, 93.15, 98.26, 114.30, 117.19, 129.90, 133.08, 133.87, 137.51, 142.64, 142.89, 155.76, 167.03. ESI-MS *m/z* calcd for $C_{29}H_{34}N_4I_2O$ ($[M]^+$): 581.2, found 581.4.

10-(8-(4-iodobenzamide)octyl))-3,6-bis(dimethylamino) acridinium iodide (**^{127}I-C_8**). DIPEA (2 eq, 20 µl, 1.1 mmol) was added to a solution of **10** (0.29 g, 0.56 mmol) *in* DMF (6 mL) and the solution was kept stirring for 30 min at 0ºC. Thereafter the reaction mixture was warmed to room temperature and tetrafluorophenyl 4-iodobenzoate (1.5 eq, 0.15 g, 0.38 mmol) was added and the mixture was kept stirring overnight. Solvent was removed under reduced pressure and the dry residue purified by column chromatography on aluminium oxide ($MeOH:CH_2Cl_2$; 1:9) to give **^{127}I-C_8** as a red solid (0.07 g, 0.11 mmol, 20%). ^{1}H NMR (300 MHz, $CDCl_3$, Me_4Si) δ 1.34-1.43 (m, 6H, C*H₂*), 1.61 (m, 4H, C*H₂*), 1.85 (m, 2H, C*H₂*), 3.23 (s, 12H, C*H₃*), 3.38 (m, 2H, C*H₂*), 4.57 (m, 2H, C*H₂*), 6.40 (s, 2H, C*H*), 6.97 (d, 2H, C*H*), 7.58 (d, 2H, C*H*), 7.73-7.78 (m, 4H, C*H*), 8.53 (s, 1H, C*H*). ^{13}C NMR (300 MHz, $CDCl_3$, Me_4Si) δ 25.99, 26.48, 26.69, 28.70, 29.03, 40.05, 41.10, 48.25, 93.10, 93.96, 114.36, 117.31, 129.82, 133.01, 133.28, 134.40, 137.35, 142.94, 155.84, 166.74. ESI-MS *m/z* calcd for $C_{32}H_{40}N_4OI_2$ ($[M]^+$): 623.2, found 623.6.

General procedure for the synthesis of tributyltin precursors

A mixture of **AO-iodobenzamides, ^{127}I-C$_3$**, **^{127}I-C$_5$** or **^{127}I-C$_8$,** and $(Sn(Bu)_3)_2$ (2.4 eq) in dry DMF (3-4 ml) was refluxed overnight under nitrogen atmosphere in the presence of a catalytic amount of $PdCl_3(PPh_3)_2$ (0.1 eq). The amount of reagents used and reaction yields are shown in **Table S1**. Solvent was removed under reduced pressure and the dry residue obtained was purified by alumina column chromatography (methanol:CH_2Cl_2; 1:9) to give the tributyltin precursors (**Sn-C$_3$**, **Sn-C$_5$** and **Sn-C$_8$**) as dark red solids.

Table S1. Reaction conditions for the synthesis of Sn-C$_3$, Sn-C$_5$ and Sn-C$_8$, and respective yields

Tributyltin precursor	AO-iodo benzamides	DMF	$(Sn(Bu)_3)_2$ 2.4 eq	$PdCl_3(PPh_3)_2$ (0.1 eq).	Reaction yields
**Sn-C$_3$**	**^{127}I-C$_3$** 64.0 mg,0.12 mmol	3 ml	82.0µL, 0.29mmol	8.56mg, 12.32µmol	0,046mmol 38%
**Sn-C$_5$**	**^{127}I-C$_5$** 44.0 mg,0.078 mmol	4 ml	48.1µL, 0.17mmol	5.08mg, 7.23µmol	0,062mmol 79%
**Sn-C$_8$**	**^{127}I-C$_8$** 50.0 mg,0.080 mmol	4 ml	53.7µL, 0.19mmol	5.63mg, 8.02µmol	0,047mmol 59%

10-(3-(4-tributylstannylbenzamide)propyl))-3,6-bis(dimethylamino) acridinium iodide (**Sn-C$_3$*****)*** ^{1}H NMR (300 MHz, $CDCl_3$, Me_4Si) δ 2.28 (m, 2H, C*H$_2$*), 3.12 (s, 12H, C*H$_3$*) 3.90 (t, 2H, C*H$_2$*), 4.68 (t, 2H, C*H$_2$*), 6.42 (s, 2H, C*H*), 6.81 (d, 2H, C*H*), 7.63 (d, 2H, C*H*), 7.73 (d, 2H, C*H*), 8.26 (d, 2H, C*H*), 8.54 (s, 1H, C*H*), 8.81 (1H, N*H*).

10-(5-(4-tributylstannylbenzamide)pentyl))-3,6-bis(dimethylamino) acridinium iodide (**Sn-C$_5$*****)*** ^{1}H NMR (300 MHz, $CDCl_3$, Me_4Si) δ 1.77 (m, 2H, C*H$_2$*), 1.97 (m, 4H, C*H$_2$*), 3.25 (s, 12H, C*H$_3$*), 3.58 (t, 2H, C*H$_2$*), 4.69 (t, 2H, C*H$_2$*), 6.59 (s, 2H, C*H*), 6.99 (d, 2H, C*H*), 7.53 (d, 2H, C*H*), 7.80 (d, 2H, C*H*), 8.12 (d, 2H, C*H*), 8.53 (s, 1H, C*H*), 8.63 (t, 1H, N*H*).

10-(8-(4-tributylstannylbenzamide)octyl))-3,6-bis(dimethylamino) acridinium iodide (**Sn-C$_8$*****)*** ^{1}H NMR (300 MHz, $CDCl_3$, Me_4Si) δ 1.61 (m, 8H, 4CH_2), 1.89 (m, 4H, C*H$_2$*), 3.25 (s, 12H, C*H$_3$*), 3.42 (m, 2H, C*H$_2$*), 4.57 (t, 2H, C*H$_2$*), 6.47 (s, 2H, C*H*), 7.00 (d, 2H, C*H*), 7.49 (d, 2H, C*H*), 7.84 (m, 4H, C*H*), 8.59 (s, 1H, C*H*).

General procedure for the synthesis of the radioiodinated acridines ^{125}I-C$_3$, ^{125}I-C$_3$ and ^{125}I-C$_8$

The radioiodinated acridines were prepared by iododestanylation using the tributyltin precursors, **Sn-C$_3$**, **Sn-C$_5$** and **Sn-C$_8$**. Briefly, hydrogen peroxide (50 µl, 3% w/v) was added to a mixture of the correspondent tributyltin precursor (100 µg in 50 µl of methanol), 50 µl of 1N HCl and 22-37 MBq of [^{125}I]NaI (0.6-1 mCi). The mixture was vortexed and allowed to react for 10 min at room temperature, and quenched by the addition of 100 µl of saturated $NaHSO_3$. After neutralization with a saturated $NaHCO_3$ solution (100 µl) the reaction mixture was extracted with ethyl acetate (5x100 µl). The combined organic layers were evaporated to dryness under a stream of nitrogen. The residues were dissolved in acetonitrile and purified by analytical RP-HPLC (method 1). The fractions containing the product were collected, combined, diluted in 10 ml of H_2O and subsequently loaded onto a C18 Sep-Pak cartridge (prewashed with 5 ml of methanol, followed by 2x5 ml of water). The radioiodinated product was eluted with 2 ml of ethanol. The identity of radioiodinated AO derivatives was confirmed by co-elution with reference compounds **(Table S2)**. HPLC radiochromatograms of the co-elution of purified **^{125}I-C$_5$** with non-radioactive analogue **^{127}I-C$_5$** are shown in **Figure S2** as an example.

Table S2. Labelling yields and HPLC retention time for ^{125}I-C$_3$, ^{125}I-C$_5$ and ^{125}I-C$_8$

Compound	Labelling yield (%)	t_r/min[a]
**^{125}I-C$_3$**	23.3	8.67 (8.42)
**^{125}I-C$_5$**	32.3	12.75 (12.35)
**^{125}I-C$_8$**	30.2	21.47 (21.22)

[a] The retention time for non-radioactive analogues, **^{127}I-C$_3$, ^{127}I-C$_5$ and ^{127}I-C$_8$**, are in parenthesis

By HPLC purification the radioiodinated AO derivatives were well separated from other radioactive species present in the reaction mixture, as well as from their nonpolar stannyl counterparts. Since no UV detection at the most sensitive detector setting was observed for the purified labelled compounds, it can be assumed that their specific activity was in the same range as that of the starting radioactive precursor [^{125}I]NaI (2200 Ci/mmol).

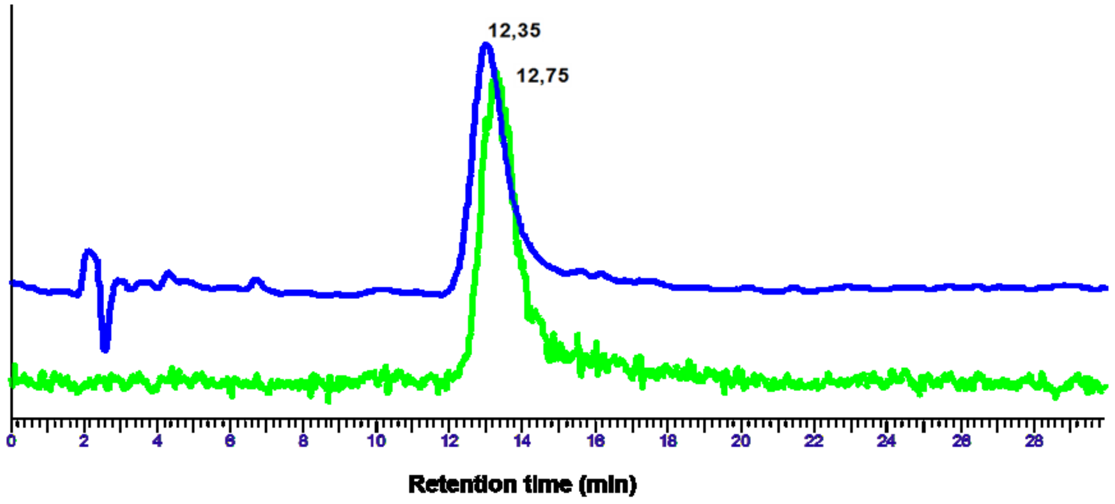

Figure S2. HPLC radiochromatograms of radioiodinated AO derivative ^{125}I-C_5 and non-radioactive analogue ^{127}I-C_5. Simultaneous (--) UV and (--) radioactivity detection.

1.2.2. Synthesis of AO-containing Re and ^{99m}Tc complexes

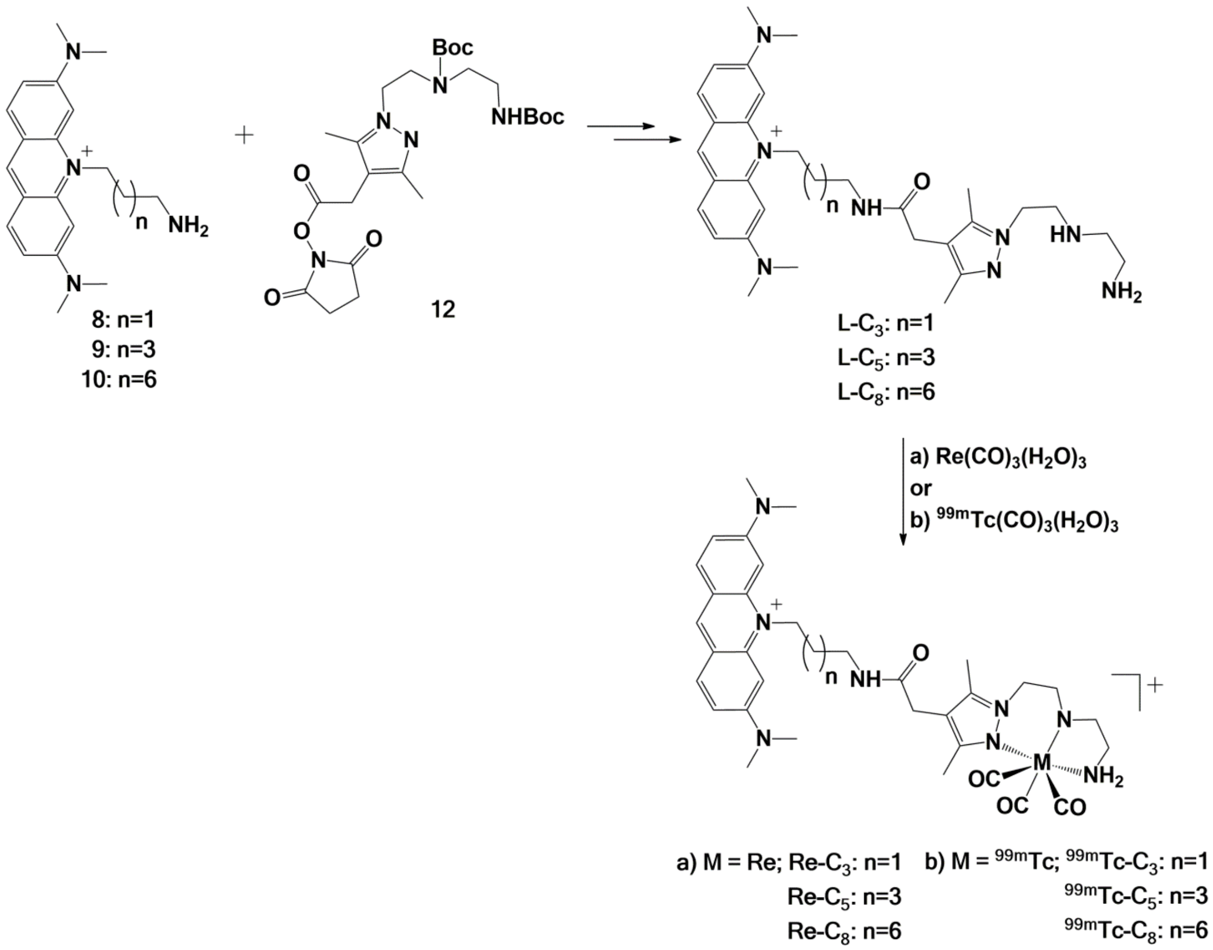

Figure S3. Synthesis of AO-containing pyrazolyl diamine chelators and corresponding metal complexes.

Pyrazolyl diamine chelators and corresponding Re(I) tricarbonyl complexes

Synthesis of the bifunctional ligands (L-C_3, L-C_5, L-C_8)

10-(3-(2-(1-(2-(2-aminoethylamino)ethyl)-3,5-dimethyl-1H-pyrazol-4 yl)acetamido)propyl) -3,6-bis(dimethylamino)acridinium) iodide (**L-C_3**).

DIPEA (1.29 mmol) was added to a solution of **8** (0.15 g, 0.43 mmol) in dry DMF (5 mL) and the mixture was stirred for 30 min. Then, compound **12** (0.86 mmol) in DMF (5 mL) was added and the mixture was stirred for 5 days at room temperature. Then, the solvent was evaporated and the residue was washed with water. The red oil obtained was dissolved in MeOH (10 mL) and cooled to 0ºC and 37% HCl (3.2 mL) was added dropwise. After 36 h stirring at room temperature the pH was adjusted to 12-14 with aqueous NaOH (3M) and the reaction mixture was extracted with $CHCl_3$. The combined organic phases were dried over $MgSO_4$ and concentrated under reduce pressure. Ligand **L-C_3** was obtained as a red oil (76 mg, 0.14 mmol, 32%) after purification by alumina column chromatography (eluent: MeOH/NH_3 100:0→99:1). R_f (1%NH_3/MeOH)=0.09. RP-HPLC, method 2, t_R=18.5 min.

^{1}H NMR (300 MHz, CD_3OD) δ 2.00 (m, 2H, CH_2), 2.09 and 2.22 (2s, 6H, CH_3), 3.08 (m, 2H, CH_2), 3.33 (m, 4H, CH_2), 3.37 (s, 12H, CH_3), 3.46 (s, 2H, CH_2), 3.58 (t, 2H, CH_2), 4.39 (t, 2H, CH_2), 4.67 (t, 2H, CH_2), 6.62 (s, 2H, C*H*), 7.27 (d, 2H, C*H*), 7.88 (d, 2H, C*H*), 8.61 (s, 1H, C*H*). ^{13}C NMR (300 MHz, MeOD) δ 9.8, 11.9, 27.0, 31.8, 38.3, 40.9, 41.1, 46.6, 48.1-49.5, 49.9, 93.4, 111.2, 115.5, 118.4, 134.4, 139.9, 143.9, 144.3, 147.9, 157.4, 174.3. ESI-MS (+) $C_{31}H_{45}N_8O^+$ (545.4) (*m/z*) 545.3 $[M]^+$.

10-(5-(2-(1-(2-(2-aminoethylamino)ethyl)-3,5-dimethyl-1H-pyrazol-4-yl) acetamido)pentyl) -3,6-bis(dimethylamino)acridinium) iodide (**L-C_5**).

DIPEA (0.68 mmol) was added to a solution of **9** (0.12 g; 0.34 mmol) in dry DMF (4 mL) and the mixture was stirred for 30 min. A solution of **12** (0.68 mmol) in DMF (4 mL) was added and the mixture was stirred for 5 days at room temperature. Then, the solvent was evaporated and the residue was washed with water. The red oil obtained was dissolved in MeOH (7 mL), cooled to 0ºC and 37% HCl (2.2 mL) was added dropwise. After 36 h stirring at room temperature the pH was adjusted to 12-14 with aqueous NaOH (3M) and the reaction mixture was extracted with $CHCl_3$. The combined organic phases were dried over $MgSO_4$ and concentrated under reduce pressure. Ligand

L-C$_5$ was obtained as a red oil (65 mg, 0.11 mmol, 32%) after purification by alumina column chromatography (eluent: MeOH/NH_3 100:0→99:1). R_f (1%NH_3/MeOH)=0.09. RP-HPLC, method 2, $\boldsymbol{t}_{\mathbf{R}}$= 20.6 min.

^{1}H NMR (300 MHz, CD_3OD) δ 1.56-1.77 (m, 4H, C*H*$_2$), 2.04 (m, 2H, C*H*$_2$), 2.91 and 3.00 (2s, 6H, C*H*$_3$), 3.08-24 (m, 6H, C*H*$_2$), 3.26 (s, 12H, C*H*$_3$), 3.36 (s, 2H, C*H*$_2$), 3.68 (m, 2H, C*H*$_2$), 4.12 (t, 2H, C*H*$_2$), 4.68 (t, 2H, C*H*$_2$), 6.63 (s, 2H, C*H*), 7.27 (d, 2H, C*H*), 7.90 (d, 2H, C*H*), 8.61 (s, 1H, C*H*). ^{13}C NMR (300 MHz, MeOD) δ 9.4, 11.9, 24.9, 28.2, 31.2, 40.3, 40.4, 40.8, 45.8-50.2, 93.7, 115.6, 115.9, 119.1, 134.5, 140.2, 144.0-144.6, 155.0, 179.5. ESI-MS (+) $C_{33}H_{49}N_8O^+$ (573.4) (*m/z*) 573.4[M]$^+$.

10-(8-(2-(1-(2-(2-aminoethylamino)ethyl)-3,5-dimethyl-1H-pyrazol-4-yl)acetamido)octyl)-3,6-bis(dimethylamino)acridinium iodide (**L-C$_8$**).

DIPEA (0.42 mmol) was added to a solution of **10** (0.08 g; 0.21 mmol) in dry DMF (11 mL). After stirring for 30 min a solution of **12** (0.21 mmol) in dry DMF (11 mL) was added and the mixture was stirred for 5 days at room temperature. Then, the solvent was evaporated and the resulting compound washed with water. The red oil obtained was dissolved in MeOH (5 mL), cooled to 0ºC and 37% HCl (1.0 mL) was added dropwise. After 36 h stirring at room temperature the pH was adjusted to 12-14 with aqueous NaOH (3M) and the reaction mixture was extracted with $CHCl_3$. The organic phases were dried over $MgSO_4$ and concentrated under reduce pressure. Ligand **L-C$_8$** was obtained as a red oil (15 mg, 0.02 mmol, 12%) after purification by alumina column chromatography (eluent: MeOH/$CHCl_3$ 20:100→100:0). R_f (1%NH_3/MeOH)= 0.15. RP-HPLC, method 2, $\boldsymbol{t}_{\mathbf{R}}$= 23.6 min.

^{1}H NMR (300 MHz, CD_3OD) δ 1.56-1.69 (m, 8H, C*H*$_2$), 2.08 (m, 2H, C*H*$_2$), 2.18 and 2.27 (2s, 6H, C*H*$_3$), 2.82 (m, 2H, C*H*$_2$), 2.98 (m, 4H, C*H*$_2$), 3.19 (m, 2H, C*H*$_2$), 3.38 (s, 12H, C*H*$_3$), 3.47 (s, 2H, C*H*$_2$), 3.70 (m, 2H, C*H*$_2$), 4.13 (m, 2H, C*H*$_2$), 4.67 (m, 2H, C*H*$_2$), 6.79 (s, 2H, C*H*), 7.36 (d, 2H, C*H*), 8.01 (d, 2H, C*H*), 8.76 (s, 1H, C*H*). ^{13}C NMR (300 MHz, MeOD) δ 8.9, 12.8, 26.6-28.3, 30.1, 33.6, 38.6, 39.9, 44.7, 46.0-48.5, 100.2, 114.2, 115.6, 117.3, 132.5, 141.1, 143.0, 143.8, 154.8, 172.9. ESI-MS (+) $C_{36}H_{55}N_8O^+$ (615.5) (*m/z*) 615.5 [M]$^+$.

General procedure for the synthesis of Re complexes

The rhenium complexes (**Re-C$_3$/Re-C$_5$/Re-C$_8$)** were obtained by reacting equimolar amounts of ligands (**L-C$_3$/L-C$_5$/L-C$_8$)** with [Re(H_2O)$_3$(CO)$_3$]Br overnight in refluxing MeOH. Thereafter the solvent was removed under reduced pressure and the residue washed with water and purified by alumina column chromatography with eluting EtOH (**Re-C$_3$)** or MeOH (**Re-C$_5$/Re-C$_8$)** to give the desired **Re** complexes as red-orange solids. **Re-C$_8$** was further purified by RP-HPLC (method 3) and was also obtained as a red-orange solid. The amount of reagents and reaction yields are shown in **Table S3**.

Table S3. Labelling conditions and yields for Re complexes Re-C$_3$, Re-C$_5$ and Re-C$_8$

Complex	bifunctional ligands	[Re(H$_2$O)$_3$(CO)$_3$]Br	MeOH (mL)	Reaction yields
**Re-C$_3$**	**L-C$_3$** 30 mg, 0.06 mmol	1 eq	5	35 mg, 0,04 mmol 78%
**Re-C$_5$**	**L-C$_5$** 43mg, 0.08 mmol	1 eq	12	28 mg, 0,03 mmol 46%
**Re-C$_8$**	**L-C$_8$** 118 mg, 0.19 mmol	1 eq	22	3 mg, 0,003 mmol 2%

Re-C$_3$. ^{1}H NMR (300 MHz, CD$_3$OD) δ 2.08 (m, 2H, C*H$_2$*), 2.30 and 2.37 (2s, 6H, C*H$_3$*), 2.61 (m, 2H, C*H$_2$*), 2.63 (m, 1H, C*H$_2$*), 2.90 (m, 2H, C*H$_2$*), 3.10 (m, 2H, C*H$_2$*), 3.38 (s, 12H, CH$_3$), 3.43 (s, 2H, C*H$_2$*), 3.54 (m, 1H, C*H$_2$*), 3.90 (br, 1H, N*H$_2$*), 4.12 (m, 1H, C*H$_2$*), 4.57 (m, 1H, C*H$_2$*), 4.73 (m, 2H, C*H$_2$*), 5.44 (br, 1H, N*H$_2$*), 6.68 (s, 2H, C*H*), 6.98 (br, 1H, N*H*), 7.26 (d, 2H, C*H*), 7.90 (d, 2H, C*H*), 8.66 (s, 1H, C*H*). ^{13}C NMR (300 MHz, MeOD) δ 10.1, 14.9, 24.8, 28.3, 31.1, 39.9, 40.3, 43.4, 48.4, 50.5, 56.2, 93.9, 114.2, 115.7, 118.8, 134.6, 143.9, 144.3, 144.5, 153.7, 157.6, 172.9, 194.3, 194.5, 198.4. ESI-MS (+) $C_{34}H_{45}N_8O_4Re$ (816.3) (*m/z*) 408.6 [M]$^{2+}$. ν_{max} (KBr) cm^{-1} 2021, 1903 (C≡O). RP-HPLC, method 2, ***t*$_R$**=21.1 min.

Re-C$_5$. ^{1}H NMR (300 MHz, CD$_3$OD) δ 2.05 (m, 4H, C*H$_2$*), 2.33 and 2.42 (2s, 6H, C*H$_3$*), 2.49 (m, 2H, C*H$_2$*), 2.60 (m, 2H, C*H$_2$*), 2.68 (m, 1H, C*H$_2$*), 2.92 (m, 2H, C*H$_2$*), 3.26 (m, 2H, C*H$_2$*), 3.38 (s, 12H, C*H$_3$*), 3.46 (s, 2H, C*H$_2$*), 3.57 (m, 1H, C*H$_2$*), 3.94 (br, 1H, N*H$_2$*), 4.13 (m, 1H, C*H$_2$*), 4.58 (m, 1H, C*H$_2$*), 4.74 (t, 2H, C*H$_2$*), 5.46 (br, 1H, N*H$_2$*), 6.69 (s,

2H, C*H*), 6.98 (br, 1H, N*H*), 7.28 (d, 2H, C*H*), 7.90 (d, 2H, C*H*), 8.71 (s, 1H, C*H*). ^{13}C NMR (300 MHz, MeOD) δ 10.4, 14.8, 25.3, 26.4, 30.1, 31.8, 33.7, 41.1, 43.4, 47.9-50.4, 56.1, 93.6, 114.4, 115.7, 119.7, 124.4, 134.6, 135.7, 143.8, 144.2, 153.3, 157.5, 173.1 ESI-MS (+) $C_{36}H_{49}N_8O_4Re$ (844.3) (*m/z*) 422.1 $[M]^{2+}$. ν_{max} (KBr) cm^{-1} 2021, 1904 (C≡O). RP-HPLC, method 2, t_R=21.8 min.

Re-C₈. ^{1}H NMR (300 MHz, MeOD) δ 1.44-1.57 (m, 8H, CH_2), 1.72 (m, 2H, CH_2), 2.32 and 2.34 (2s, 6H, CH_3), 2.53 (m, 2H, CH_2), 2.91 (m, 3H, CH_2), 3.11 (t, 2H, CH_2) 3.38 (s, 12H, CH_3), 3.55 (s, 2H, CH_2), 3.92 (m, 1H, CH_2), 4.15 (m, 2H, CH_2 and NH_2), 4.40 (m, 1H, CH_2), 4.58 (m, 2H, CH_2), 5.47 (br, 1H, NH_2), 6.80 (s, 2H, C*H*), 6.98 (br, 1H, N*H*), 7.36 (d, 2H, C*H*), 7.98 (d, 2H, C*H*), 8.75 (s, 1H, C*H*). ^{13}C NMR (300 MHz, MeOD): δ = 9.26, 9.28, 24.1, 24.2, 26.7, 27.7, 29.9-31.5, 33.1, 33.2, 40.3-41.3, 47.8, 52.3, 55.7, 93.5, 113.9, 115.5, 118.7, 130.3, 133.0, 134.5, 138.9, 143.4, 144.2, 156.8, 198.0-199.7. ESI-MS (+) $C_{39}H_{55}N_8O_4Re$ (886.4) (*m/z*) (%): 443.9 $[M]^{2+}$, 482.4 $[M]^{2+}$+K. ν_{max} (KBr) cm^{-1} 2023, 1900 (C≡O). RP-HPLC, method 3, t_R=30.5 min.

General procedure for the synthesis of ^{99m}Tc complexes: ^{99m}Tc-C$_3$, ^{99m}Tc-C$_5$, and ^{99m}Tc-C$_8$

The radioactive precursor *fac*-[^{99m}Tc(CO)$_3$(H$_2$O)$_3$]$^+$ was prepared by reaction of Na[^{99m}TcO$_4$] (3 mL) with a mixture of sodium boranocarbonate (4.5 mg), sodium tartrate (8.5 mg), sodium carbonate (7.2 mg) and sodium tetraborate (2.9 mg). After heating for 30 min at 100º the pH was adjusted to 7. The radioactive precursor *fac*-[^{99m}Tc(CO)$_3$(H$_2$O)$_3$]$^+$ (1.5 mL) was then added to a 10^{-3} M ethanolic solution of the appropriate ligand **L-C$_3$**, **L-C$_5$** or **L-C$_8$**. The reaction mixtures were then heated for 30 min at 100°C to give the corresponding **^{99m}Tc-C$_3$**, **^{99m}Tc-C$_5$** and **^{99m}Tc-C$_8$** complexes after RP-HPLC purification (method 3). RP-HPLC, **^{99m}Tc-C$_3$:** t_R=21.6 min; **^{99m}Tc-C$_5$:** t_R=21.8 min; **^{99m}Tc-C$_8$:** t_R=31.6 min.

The chemical identity of the ^{99m}Tc complexes was ascertained by comparison of their HPLC profiles with those of the corresponding rhenium complexes. HPLC radiochromatograms of the co-elution of purified **^{99m}Tc-C$_5$** complex with its corresponding rhenium complex, **Re-C$_5$** are shown in **Figure S4** as a representative example.

HPLC purification of the AO-containing ^{99m}Tc-complexes removed the excess of the pyrazolyl-diamine chelators, which could not be detected at the most sensitive UV detector setting. Hence, it can be considered that their specific activity is in the same range as that of the starting Na^{99m}TcO$_4$ (ca. 100,000 Ci/mmol for ^{99m}Tc obtained from a ^{99}Mo/^{99m}Tc generator undergoing daily elution[5]).

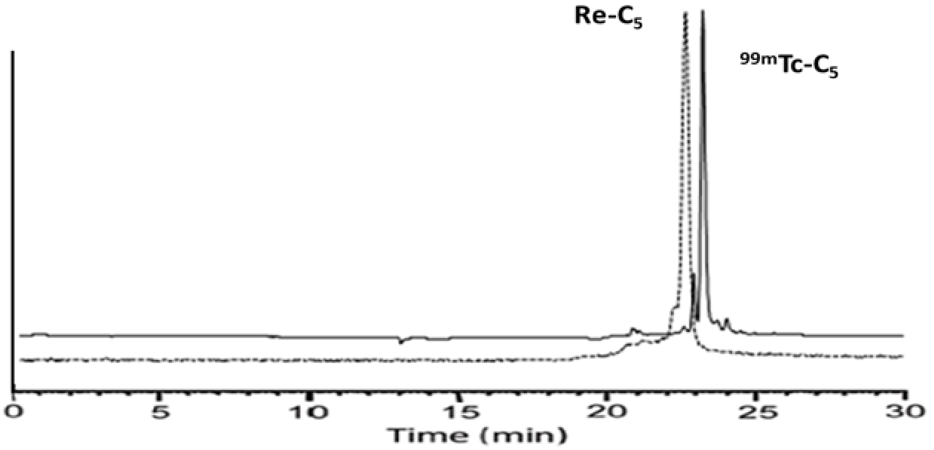

Figure S4. HPLC radiochromatograms of purified ^{99m}Tc-C$_5$ complex and corresponding rhenium complex, Re-C$_5$. Simultaneous (···) UV and (-) radioactivity detection.

1.4. *In vitro* stability studies

Radiochemical stability of the radioiodinated derivatives (**^{125}I-C$_3$**, **^{125}I-C$_5$** and **^{125}I-C$_8$**) and ^{99m}Tc complexes (**^{99m}Tc-C$_3$**, **^{99m}Tc-C$_5$** and **^{99m}Tc-C$_8$**) was assessed by RP-HPLC analysis at several time points following incubation in Tris-HCl buffer and in DMEM cell medium at 37ºC. The HPLC radiochromatograms of **^{125}I-C$_5$** (method 1) and **^{99m}Tc-C$_5$** complex (method 2) following incubation in DMEM cell medium are shown in **Figures S5** and **S6**, respectively, as representative examples.

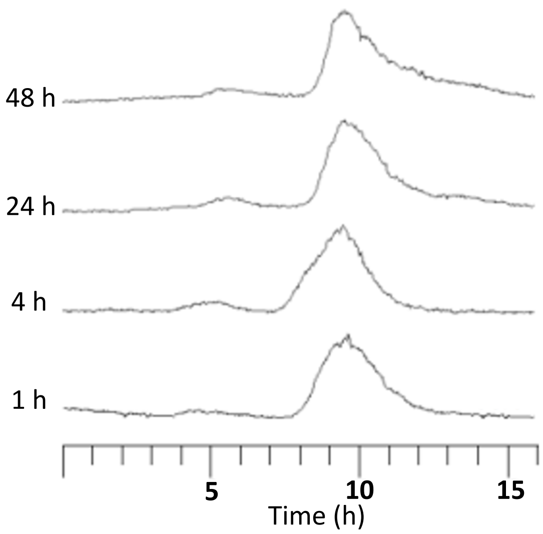

Figure S5. In vitro stability of ^{125}I-C$_5$. Radiochromatograms of ^{125}I-C$_5$ following incubation in DMEM medium at 37ºC.

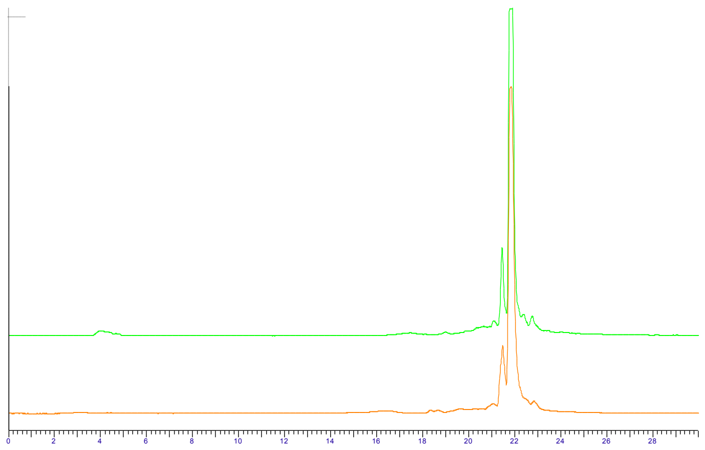

Figure S6. In vitro stability of ^{99m}Tc-C$_5$. Radiochromatograms of ^{99m}Tc-C$_5$ complex (---), following 4h incubation (---) in DMEM medium at 37ºC.

2. Spectroscopic evaluation of the DNA binding ability

2.1. UV-Vis absorption spectra

UV-Vis spectra were measured in all systems in order to correct the fluorescence emission spectra for reabsorption and inner-filter effects. [6-8] Although these spectra were not used for the determination of binding parameters, they give a clear indication of the strong interaction between all compounds and DNA, due to the occurrence of hypochromism and red shifts in the absorption bands. **Figures S7-S10** show the UV-Vis absorption spectra measured for the different tested compounds in the presence of increasing amounts of CT-DNA. **Table S4** summarizes the observed effects.

Table S4. UV-Vis absorption bands and absorption values in the absence and presence of DNA

	λ_{max} (Abs), no DNA	λ_{max} (Abs), with DNA	$\Delta\lambda$ (nm)
^{127}I-C$_3$	444 (0.103)	448 (0.071)	4
	503.5 (0.135)	508 (0.129)	4.5
^{127}I-C$_5$	479.5 (sh, 0.190)	483.5 (sh, 0.124)	4
	499.5 (0.259)	505 (0.168)	5.5
^{127}I-C$_8$	499 (0.14)	505 (0.10)	6
Re-C$_3$	497 (0.065)	501 (0.408)	4
Re-C$_5$	478 (sh, 0.213)	481 (sh, 0.170)	3
	498 (0.261)	502 (0.213)	4

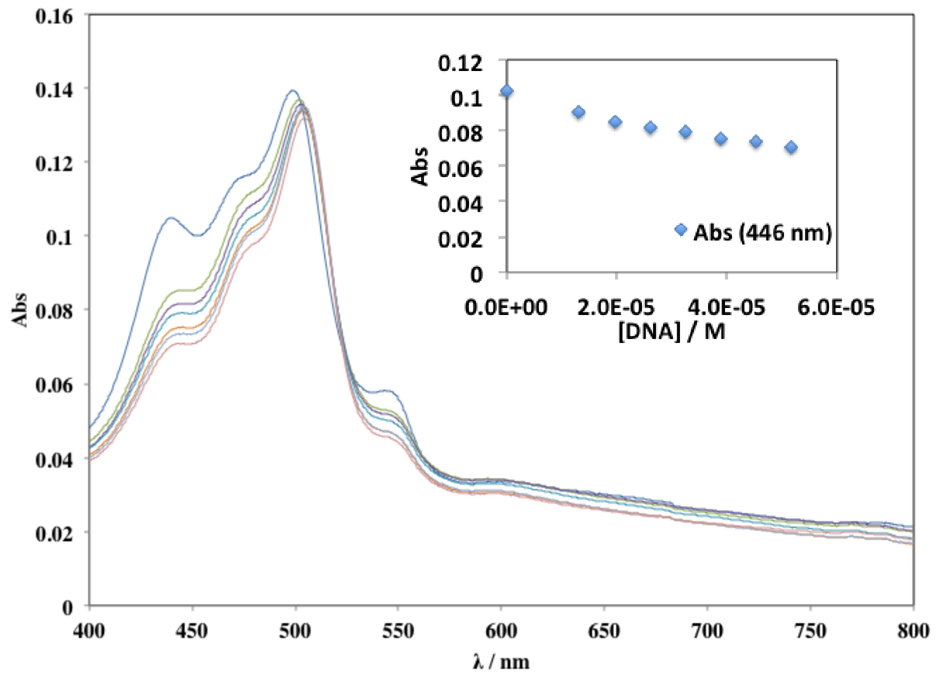

Figure S7. UV-Vis absorption spectra of [127]I-C_3. Spectra measured for solutions containing [127]I-C_3 (ca. 7µM) and increasing amounts of CT-DNA (ca. 3.3mM). Inset: Variation of the absorption at 446 nm *vs.* [DNA]. Path length 1 cm.

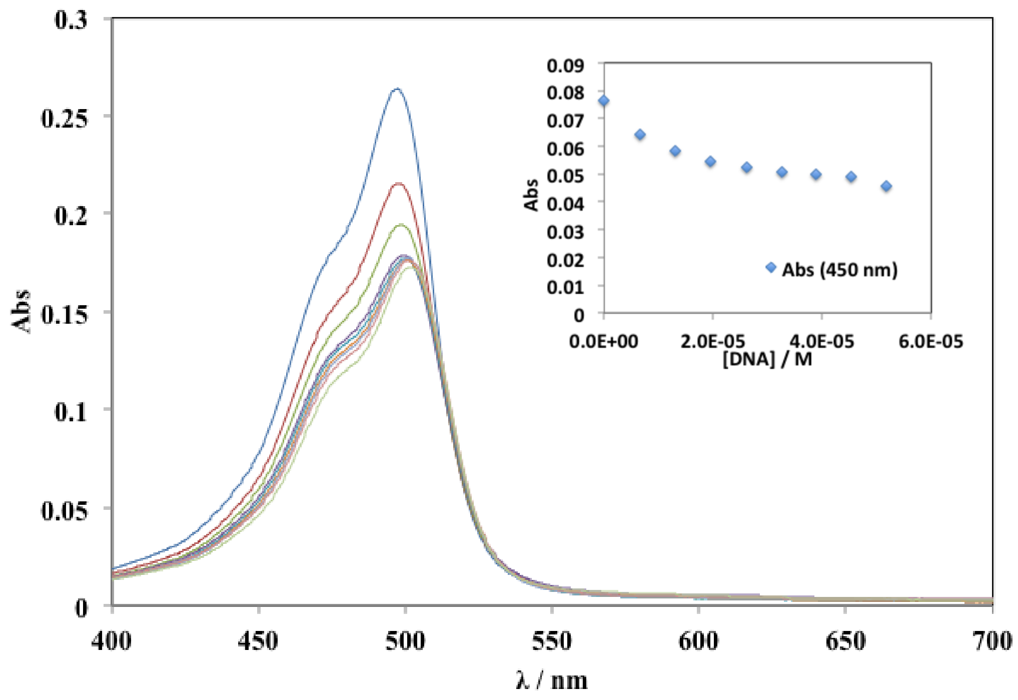

Figure S8. UV-Vis absorption spectra of [127]I-C_5. Spectra measured for solutions containing [127]I-C_5 (ca. 4µM) and increasing amounts of CT-DNA (ca. 3.3mM). Inset: Variation of the absorption at 450 nm *vs.* [DNA].

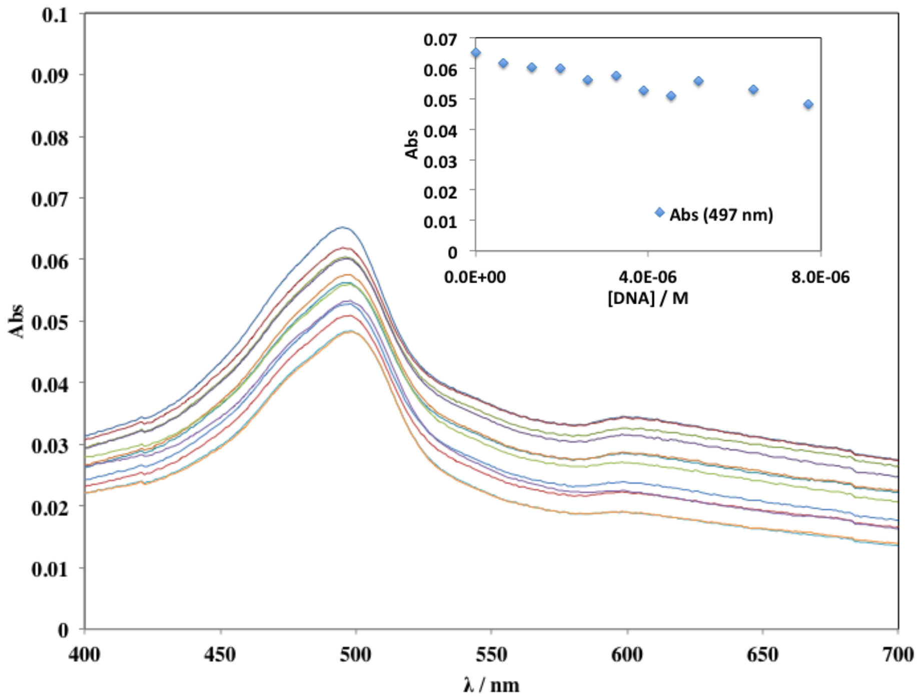

Figure S9. UV-Vis absorption spectra of Re-C_3. Spectra measured for solutions containing complex Re-C_3 (ca. 1µM) and increasing amounts of CT-DNA (ca. 3.3mM). Inset: Variation of the absorption at 497 nm *vs.* [DNA].

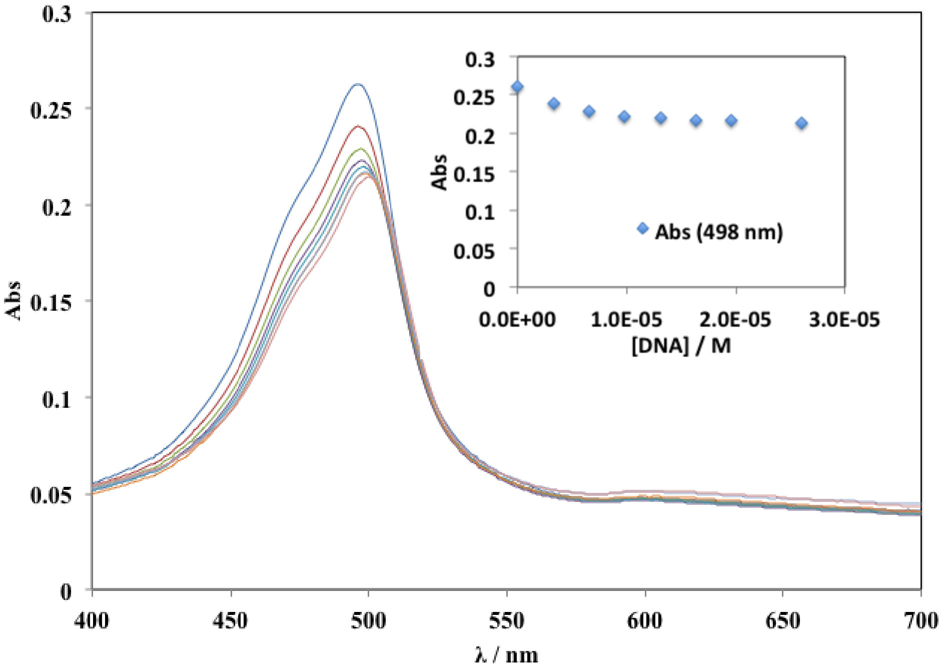

Figure S10. UV-Vis absorption spectra of Re-C_5. Spectra measured for solutions containing complex Re-C_5 ca. 8µM) and increasing amounts of CT-DNA (ca. 3.3mM). Inset: Variation of the absorption at 498 nm *vs.* [DNA].

2.2. Fluorescence titrations

The fluorescence binding experiments with CT-DNA were performed in a quartz cuvette of 1 cm path length. Bandwidth was typically 5 nm in both excitation and emission. Fluorescence titrations were done in which increasing amounts of a CT-DNA solution (ca. 3.3mM) were added to the solution containing the fluorescent compounds (see **Table S5** for conditions, such as concentrations and excitation wavelengths). The concentrations were selected in order to have absorbance values below 0.2 at the excitation and emission wavelengths. Excitation wavelengths were chosen near isobestic points to avoid strong changes in the absorbance values. Blank fluorescence spectra (containing everything except the fluorophore) were measured and subtracted from each sample's emission spectra.

Table S5. Experimental conditions used in fluorescence titrations

**Compound**	**^{127}I-C$_3$**	**^{127}I-C$_5$**	**^{127}I-C$_8$**	**Re-C$_3$**	**Re-C$_5$**
Concentration/μM	7	4	3.3	1	4
DNA:compound ratio	0-7	0-13	0-15	0-10	0-13
λ_{exc} /nm	510	510	505	510	510
λ_{em} /nm	529	532	523	525	526

Two types of data treatment were done. According to the Kaminoh model [9] the data was fitted to eqn. (1).

$$I = (I_0 + K\,[\mathrm{DNA}]\,I_{sat})/(1 + K\,[\mathrm{DNA}]) \qquad (1)$$

As the concentration of the fluoropohore and its emission intensity (I_0) are known, the value in saturation conditions, I_{sat}, can be calculated from the representation of I *vs.* [DNA].

According to McGhee von Hippel model [10] the concentration of the free probe in each sample (C_F) can be calculated using eqn. (2), where C_T is the total concentration of the probe and P is the ratio of the observed fluorescence intensity of the bound probe to that of the free probe.

$$C_F = C_T(I/I_0 - P)/(1 - P) \quad (2)$$

The value of P is the y-intercept from the plot of I/I_0 *vs.* 1/[DNA], I and I_0 are the fluorescence intensities of the probes in the presence or absence of DNA. The amount of bound probe (C_B) at any concentration is given by C_T - C_F. The binding constant (K) and the binding site size (n) in base pairs were obtained from the plot of r/C_F *vs.* r, where $r = C_B$/[DNA] using eqn. (3).

$$r/C_F = K(1-nr)[(1-nr)/[1-(n-1)r]]^{n-1} \quad (3)$$

Figures S11-S15 show the fluorescence titration spectra for the different compounds, and the respective fittings obtained using both models.

a)

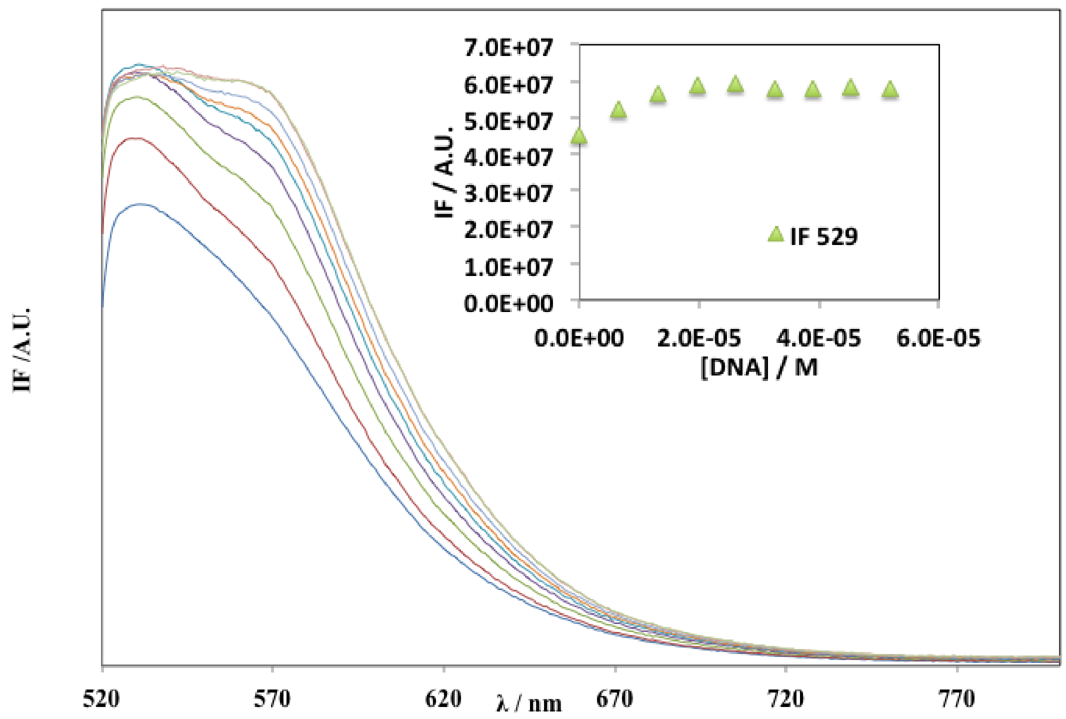

b)

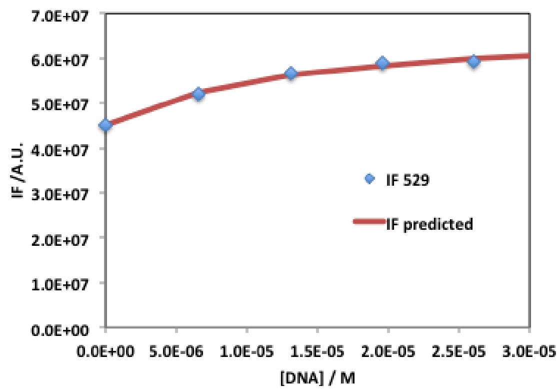

c)

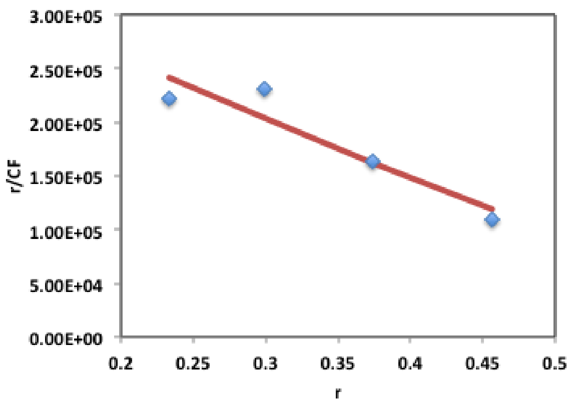

Figure S11. Fluorescence emission spectra of ^{127}I-C_3. a) Fluorescence emission spectra measured for solutions containing ligand ^{127}I-C_3 (ca. 7µM) and increasing amounts of CT-DNA, after subtraction of blank emission spectra. Inset: Variation of the fluorescence intensity at 529 nm after correction of inner filter effects. Excitation at 510 nm. b) Kaminoh model fitting of the emission intensity of ligand ^{127}I-C_3 at 529 nm (R^2 = 0.995, see table 1 for other parameters). c) McGhee von Hippell model fitting of the emission intensity of ligand ^{127}I-C_3 at 529 nm (R^2 = 0.872, see table 1 for other parameters).

a)

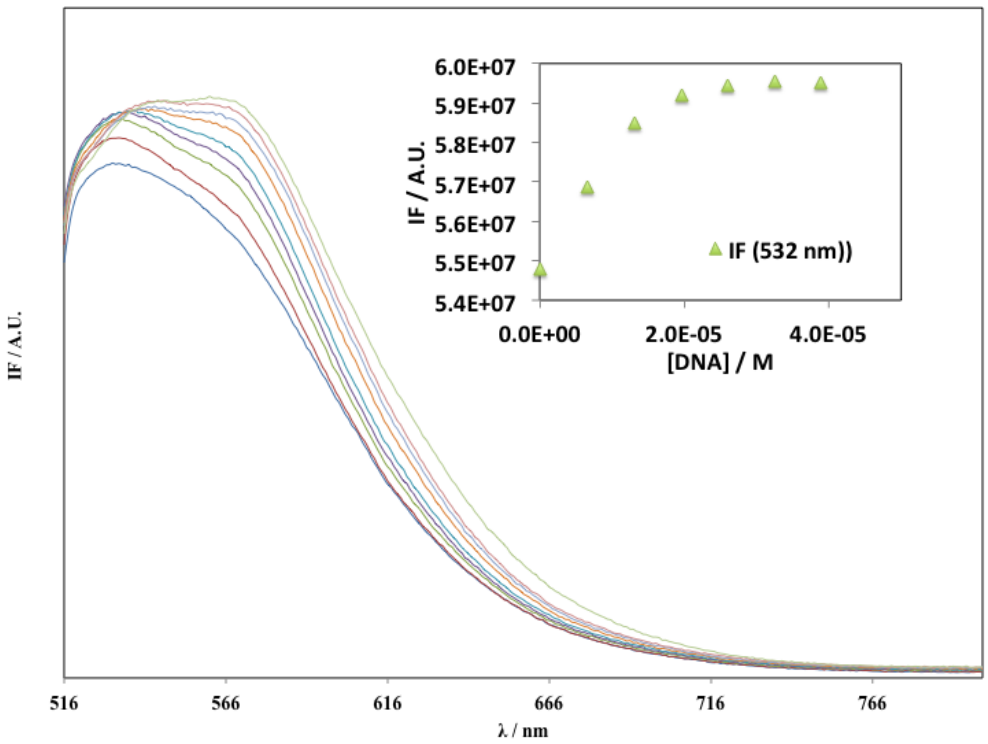

b)

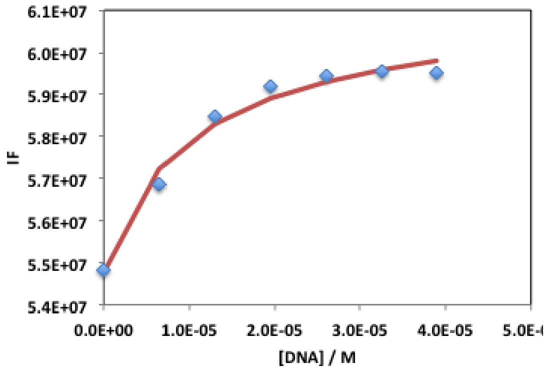

c)

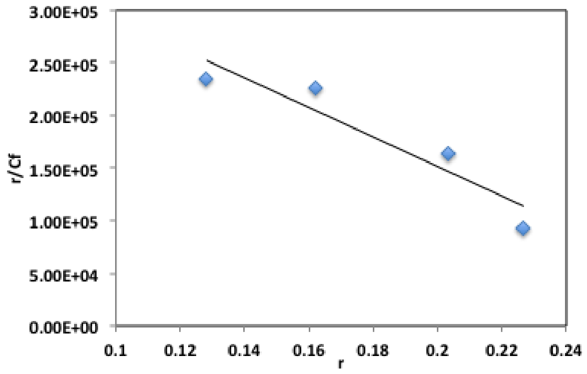

Figure S12. Fluorescence emission spectra of ^{127}I-C_5. a) Fluorescence emission spectra measured for solutions containing ^{127}I-C_5 (ca. 4μM) and increasing amounts of CT-DNA, after subtraction of blank emission spectra. Inset: Variation of the fluorescence intensity at 532 nm after correction for inner filter effects. Excitation at 510 nm. b) Kaminoh model fitting of the emission intensity of ligand ^{127}I-C_5 at 532 nm (R^2 = 0.983, see table 1 for other parameters). c) McGhee von Hippel model fitting of the emission intensity of ligand ^{127}I-C_5 at 532 nm (R^2 = 0.864, see table 1 for other parameters).

a) b)

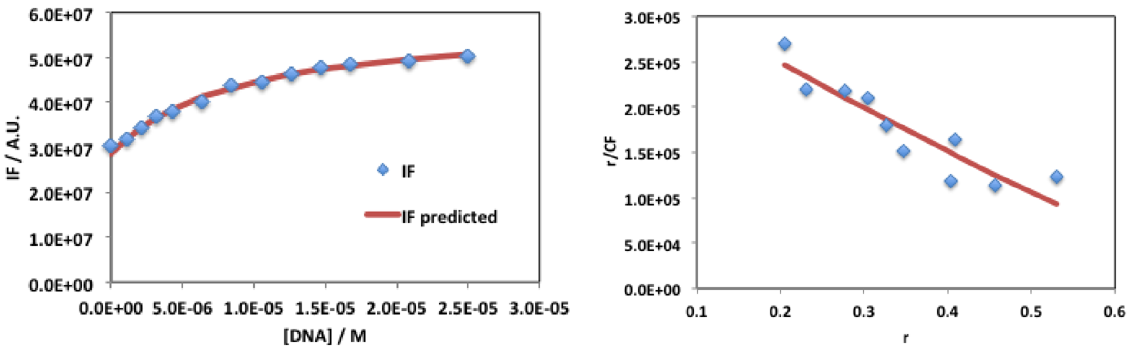

Figure S13. Model fitting of emission spectra of ^{127}I-C_8. a) Kaminoh model fitting of the emission intensity of ligand ^{127}I-C_8 at 526 nm (R^2 = 0.996, see table 1 for other parameters). b) McGhee von Hippell model fitting of the emission intensity of ligand ^{127}I-C_8 at 526 nm (R^2 = 0.838, see table 1 for other parameters).

a)

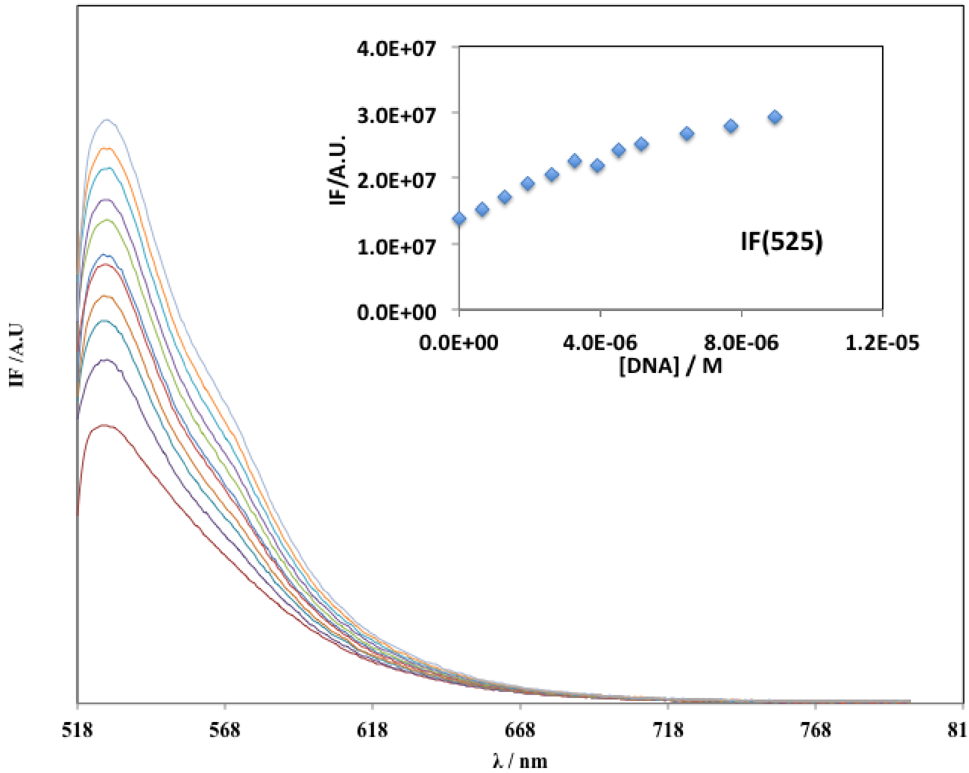

b)

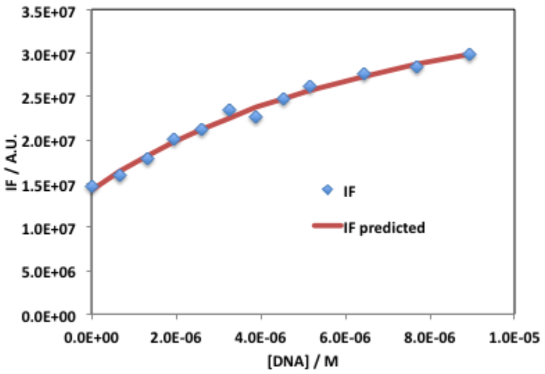

c)

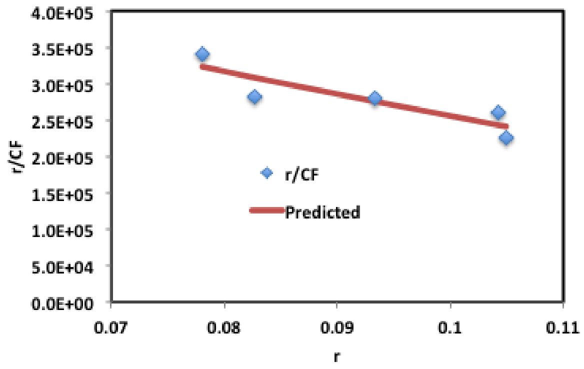

Figure S14. Fluorescence emission spectra of complex Re-C_3. a) Fluorescence emission spectra measured for solutions containing Re-C_3 (ca. 1µM) and increasing amounts of CT-DNA, after subtraction of blank emission spectra. Inset: Variation of the fluorescence intensity at 525 nm after correction for inner filter effects. Excitation at 510 nm. b) Kaminoh model fitting of the emission intensity of complex Re-C_3 at 525 nm (R^2 = 0.990, see table 1 for other parameters). c) McGhee von Hippel model fitting of the emission intensity of complex Re-C_3 at 525 nm (R^2 = 0.769, see table 1 for other parameters).

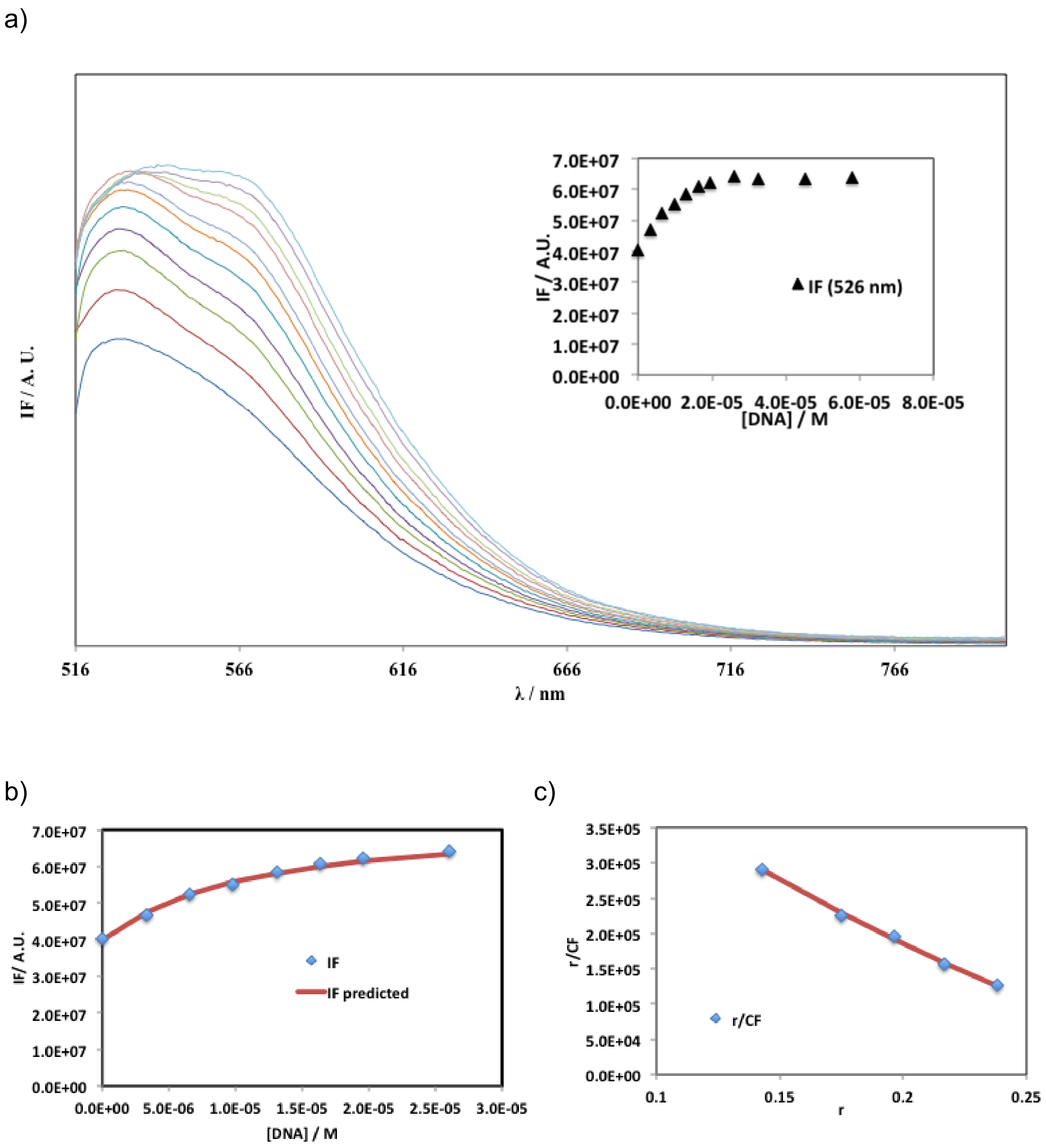

Figure S15. Fluorescence emission spectra of complex Re-C_5. a) Fluorescence emission spectra measured for solutions containing complex Re-C_5 (ca. 4.0μM) and increasing amounts of CT-DNA, after subtraction of blank emission spectra. Inset: Variation of the fluorescence intensity at 526 nm after correction for inner filter effects. Excitation at 510 nm. b) Kaminoh model fitting of the emission intensity of complex Re-C_5 at 526 nm (R^2 = 0.989, see table 1 for other parameters). c) McGhee von Hippel model fitting of the emission intensity of complex Re-C_5 at 526 nm (R^2 = 0.997, see table 1 for other parameters).

2.3 Circular Dichroism

Circular dichroism (CD) is widely used to study the affinity and binding modes of small molecules to biomolecules, particularly DNA. [11,12] When the compounds are not chiral and thus present no CD signal, their association with the right-handed DNA helix may give rise to induced CD spectra (ICD) in the range where they absorb. Moreover, DNA is chiral due to being placed within the framework of the chiral sugar–phosphate backbone, producing a characteristic CD spectrum in the 200–300 nm range. Therefore, changes in the CD signal in this spectral range indicate DNA conformational changes. For the compounds studied the focus was on the induced CD signal above 300 nm, although small conformational changes were also observed whenever spectra were measured below 300 nm.

The spectra measured for the different compounds are presented in **Figure S16**. In some cases (**^{127}I-C$_3$** in which a cell with 1 cm of path length was used, and **Re-C$_3$** in which the complex concentration was much lower) the ICD signal is weak but this was due to the low solubility of the compound, which precluded the use of higher concentrations that are needed for the observation of more intense ICD signals. **Table S6** resumes the data.

Table S6. CD bands (and signal) observed upon interaction with DNA

^{127}I-C$_3$	^{127}I-C$_5$	^{127}I-C$_8$	Re-C$_3$	Re-C$_5$
456 (+)				473 (-)
499 (-)	499 (sh, +)	480 (-)	492 (-)	490 (sh, -)
516 (+)	511 (+)	510 (+)	513 (+)	513 (+)

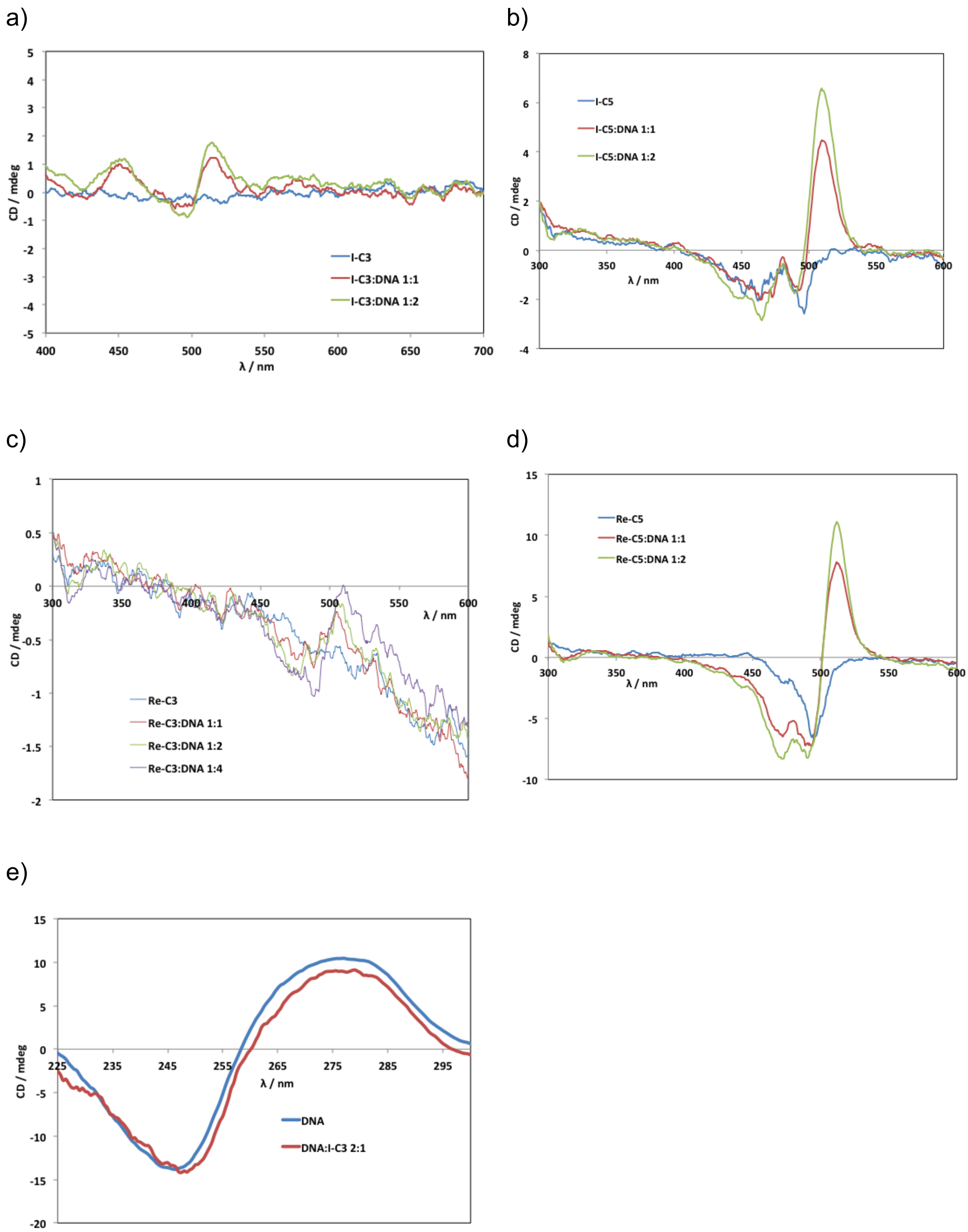

Figure S16. Circular dichroism spectra. a) ^{127}I-C$_3$ ([^{127}I-C$_3$]=72µM) in the absence and presence of DNA (ratios DNA: ^{127}I-C$_3$=1 and 2). Path length used was 1 cm; b) ^{127}I-C$_5$ ([^{127}I-C$_5$]=20µM) in the absence and presence of DNA (ratio DNA: ^{127}I-C$_5$=1 and 2). Path length used was 2 cm; c) Re-C$_3$ ([Re-C$_3$]=5µM) in the absence and presence of DNA (ratio DNA: Re-C$_3$=1, 2 and 4). Path length used was 2 cm; d) Re-C$_5$ ([Re-C$_5$]=40µM) in the absence and presence of DNA (ratio DNA:Re-C$_5$=1 and 2). Path length used was 2 cm: e) DNA ([DNA]=72µM) in the absence and presence of ^{127}I-C$_3$ (ratio DNA: ^{127}I-C$_3$= 2). Path length used was 1 cm.

3.1. Molecular docking validation

The molecular docking simulation protocol were validated by first docking the bis-intercalated anthracycline drug present in the NMR solution structure of the d(ACGTACGT)$_2$ sequence (PDB code 1AL9) [13]. The top-ranked pose revealed the anthracycline bis-intercalated in ds-DNA structure in a similar pose to that of the NMR structure solution, with a ligands all atoms root-mean-square deviation (RMSD) of 1.18 Å (**Figure S17**) and demonstrating the effectiveness of the chosen methodology.

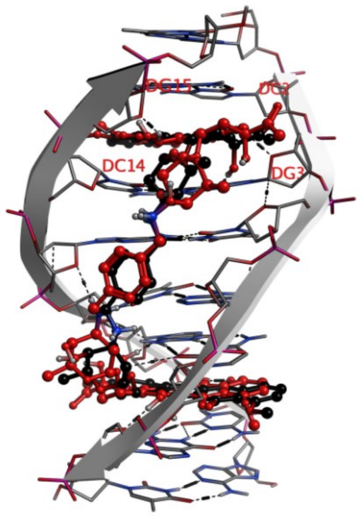

Figure S17. View of the predicted top-ranked pose upon molecular docking of the bis-intercalating anthracycline drug ligand present in the NMR solution structure PDB code 1AL9. NMR solution structure ligand showed in black and docked ligand showed in red.

3.2. Molecular Dynamics simulations

The structures of the radioiodinated AO derivatives (**^{125}I-C$_3$, ^{125}I-C$_5$** and **^{125}I-C$_8$**) were optimized and equilibrated using multiple minimization steps, followed by 1 ns NVT molecular dynamics (MD) run and a final unconstrained 50 ns MD production in an isothermal-isobaric ensemble. According to the all atoms root-mean-square deviation (RMSD) values, the equilibrium of the ds-DNA structure is quickly reached and remains stable during the entire simulation. However, the equilibrium of the AO in their binding site is only reached after the first 20 ns of the MD production. As such, only the MD data beyond this point was used to monitor the distance between the ^{125}I radionuclide and helical axis of the ds-DNA structure. The RMSD in MD trajectories of all atoms ds-DNA structure in complex with the AO derivatives are stable all over the simulation, with RMSD values around 2 Å for **^{125}I-C$_3$** and **^{125}I-C$_8$** and around 1.5 Å for **^{125}I-C$_5$** (**Figure S18a**).

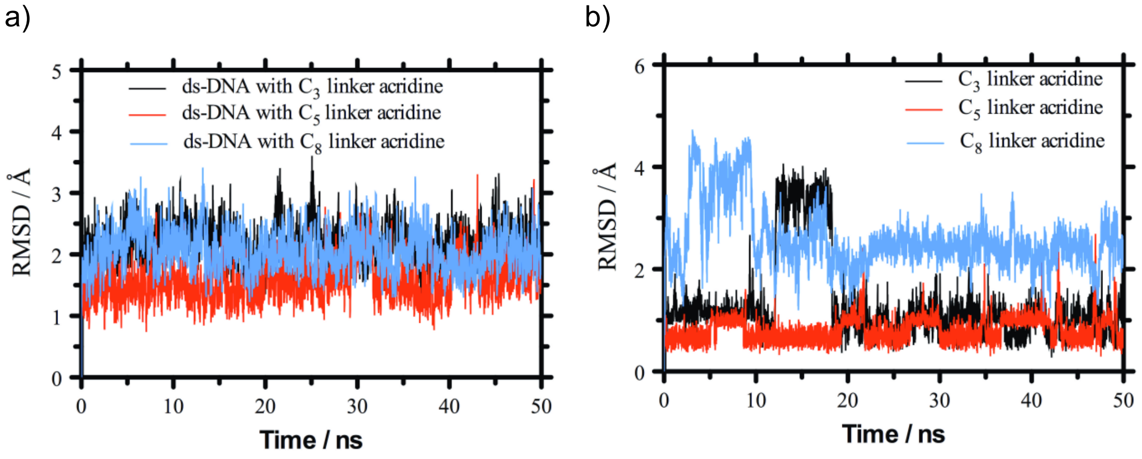

Figure S18. RMSD along the 50 ns MD trajectory of a) 8-mer ds-DNA sequence atoms and b) acridine ligands atoms. Structures at the beginning of MD production were used as reference.

The RMSD values of the acridine ligands atoms stabilize after the first 20 ns of the MD production, with values of ca. 1.0 Å for **^{125}I-C$_3$** and **^{125}I-C$_5$** and of ca. 2.5 Å for linker**^{125}I-C$_8$** (**Figure S18b**). The values are similar to the all atoms RMSD values of the ds-DNA structure, indicating that acridine derivatives are preserved well in the binding site of each model. This was also confirmed by the visual inspection of the MD runs.
As verified in the top-ranked molecular docking poses, during the MD simulations the acridine derivatives are stabilized by π-π stacking interactions with the nucleobases and it is worth noting that the acridine aromatic ring remains stable between GC base pairs during all simulation. Although, acridine side chains showed high flexibility and amplitude of movement and aid the complex stabilization mainly by performing H-bonding with phosphate backbone, nucleobases and/or water molecules (**Figure S19**).

In Figure S18, the ^{125}I distance to ds-DNA helical axis is plotted along the 50 ns MD simulation. Additionally, time relevant MD snapshots are presented to depict the dynamics of the ^{125}I radionuclide towards the ds-DNA structure in the last 30 ns of the simulation. In the last 30 ns of the MD simulation (**Figure S20**), the average distances between ds-DNA helical axis and ^{125}I in acridine side chains range between 9.9 Å and 14.8 Å. As such the average distance between ^{125}I and the ds-DNA helical axis is highly dependent of the acridine side-chain length and corroborates the previous docking results. Moreover, the influence of ^{125}I in the ds-DNA structure is affected not only by the distance to the helix, but also by radionuclide contact time. Therefore, it is expected that acridine derivatives with side chains with higher amplitude of movement exhibit lower contact time between ^{125}I and the ds-DNA. In fact, as shown in **Figure S20**, the amplitude of movement is also highly dependent of the side chain length with values of 7.4 Å for **^{125}I-C$_3$**, 10.4 Å for **^{125}I-C$_5$** and 14.8 Å for **^{125}I-C$_8$**.

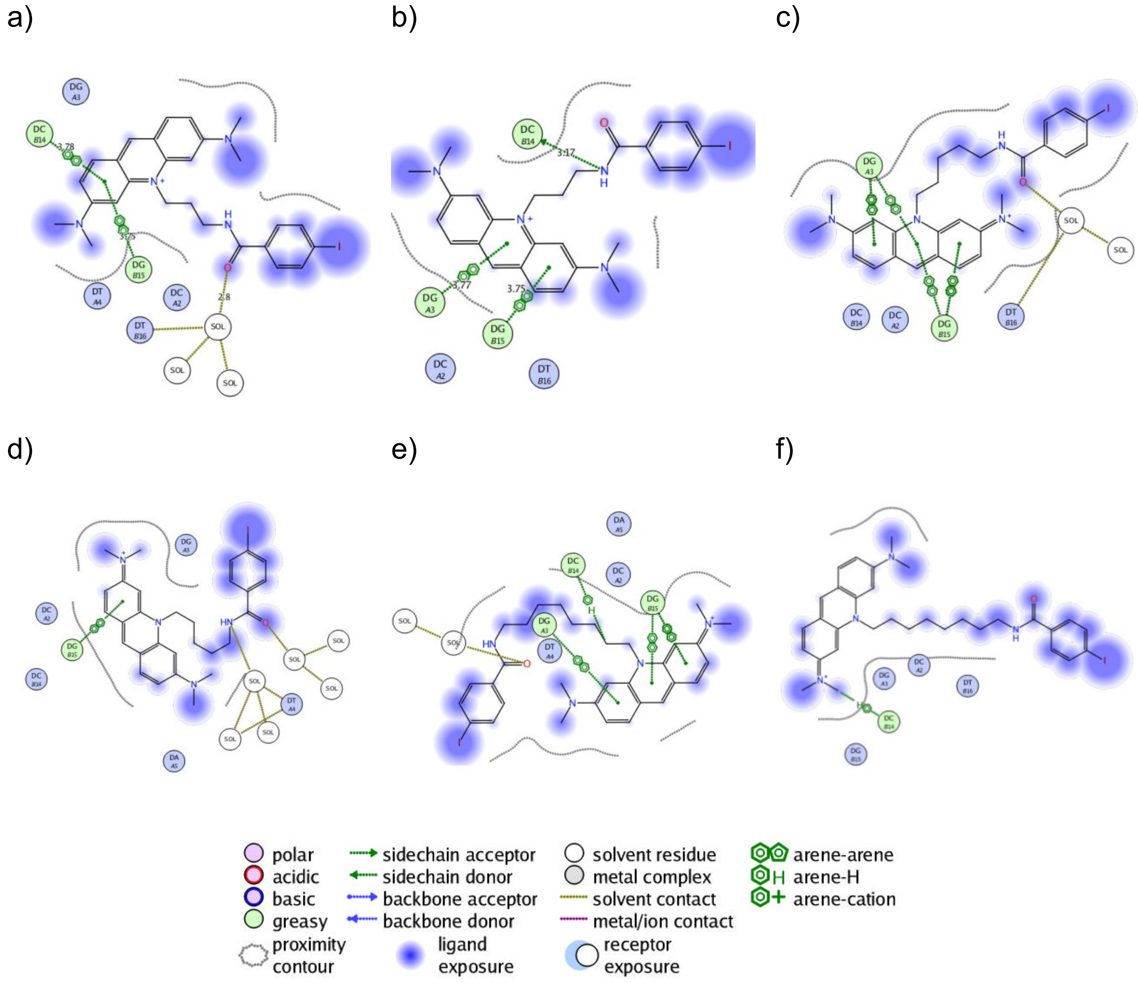

Figure S19. Interactions in the complex between the 8-mer ds-DNA and acridine derivatives in relevant snapshots of MD simulation. a) Snapshot of ^{125}I-C$_3$ at 26.6 ns; b) Snapshot of ^{125}I-C$_3$ at 39.0 ns; c) Snapshot of ^{125}I-C$_5$ at 25.3 ns; d) Snapshot of ^{125}I-C$_5$ at 42.9 ns; e) Snapshot of ^{125}I-C$_8$ at 30.3 ns; f) Snapshot of C$_8$ ^{125}I-C$_8$ at 41.7 ns.

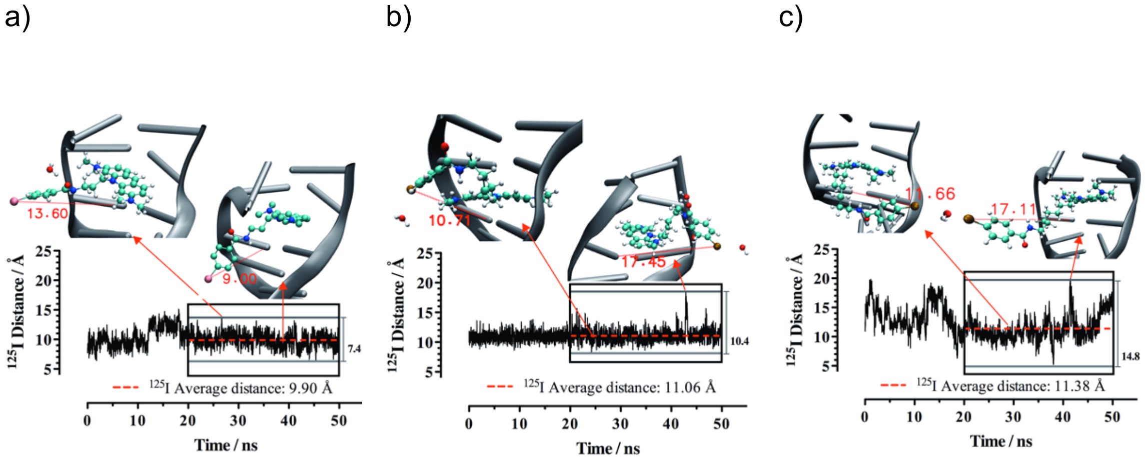

Figure S20. Distance between ^{125}I radionuclide and the ds-DNA helical axis along the 50 ns of MD simulation. Detail of the average distance and amplitude of displacement in the last 30 ns of the MD simulation as well as time relevant MD snapshots. a) ^{125}I-C_3 snapshot at 26.6 and 39.0 ns; b) ^{125}I-C_5 snapshot at 25.3 and 42.9 ns; c) ^{125}I-C_8 snapshot at 30.3 and 41.7 ns.

4. DNA damage

4.1. Estimation of DSBs

To quantify the ability of the different compounds to induce DNA double strand breaks (DSB), the yield of DBS per decay was determined. First, the number of accumulated radioactive decays was calculated from equation 4, where A_0 represents the number of disintegrations per minute (dpm) per unit of volume cm^3 and $T_{1/2}$ the semi disintegration period of the isotope in question

$$\text{N decay/cm}^3 = A_0\left(1 - EXP^{\frac{-ln2\times t}{T_{1/2}}}\right) \times \frac{T_{1/2}}{ln2} \qquad (4)$$

The number of double strand breaks (NDSB) per plasmid were calculated next. NDSB was estimated based on the relative proportion Lin of linear plasmids [14]:

$$\text{NDSB} = \text{Lin}/(1-\text{Lin}) \qquad (5)$$

The average number of DSB as a result of accumulated decays can be obtained by plotting DSB against total accumulated decays per volume and calculating the slope.
The slope of the linear regression reflects the DSB yield (YDSB) expressed as the number of DSB generated in one DNA molecule per decay of per ml. The reciprocal of the slopes represents D_0, a parameter that reflect the number of decays per ml required to form one DSB in one plasmid DNA molecule.
The slope when multiplied by the concentration of plasmid DNA in total number of plasmid DNA molecules per ml, gives the yield of DSB per decay [14,15]:

$$Y_{DSB} = \frac{[ADN]}{D_0} \qquad (6)$$

4.2. DNA interaction studies by gel electrophoresis

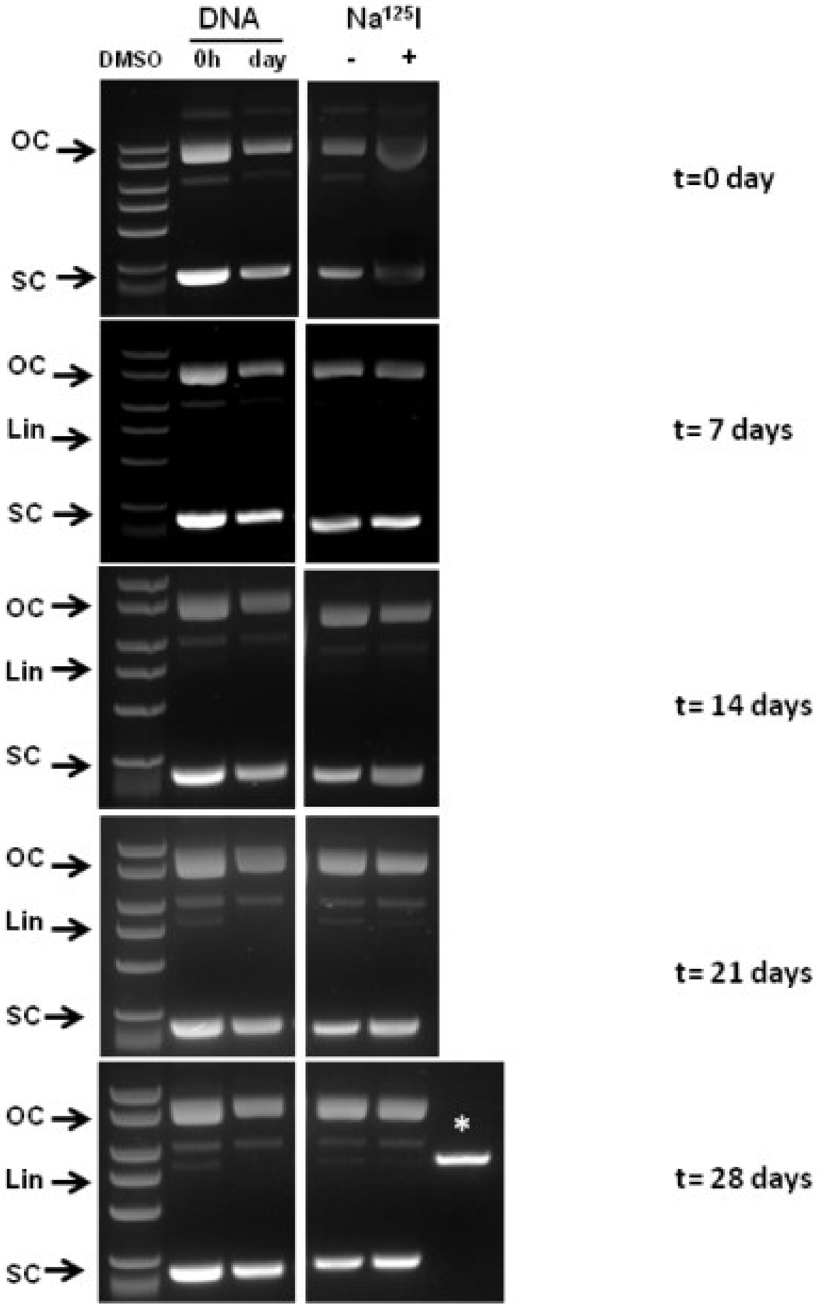

Figure S21. Cleavage of supercoiled ϕX174 DNA by Na^{125}I. Incubation of DNA with ~40 μCi of Na^{125}I was performed for 28 days at 4°C in Tris.HCl buffer (pH 7.4) in the presence or absence of DMSO (0.2M). SC, OC and Lin are supercoiled, open circular and linear forms of DNA, respectively. "DNA 0h" is the DNA control without any incubation and "DNA day" is the DNA control without compound but incubated at 4°C in Tris.HCl buffer (pH 7.4) for the same period as the ^{125}I compounds. * is a control of linearized plasmid (incubated with *XhoI*)

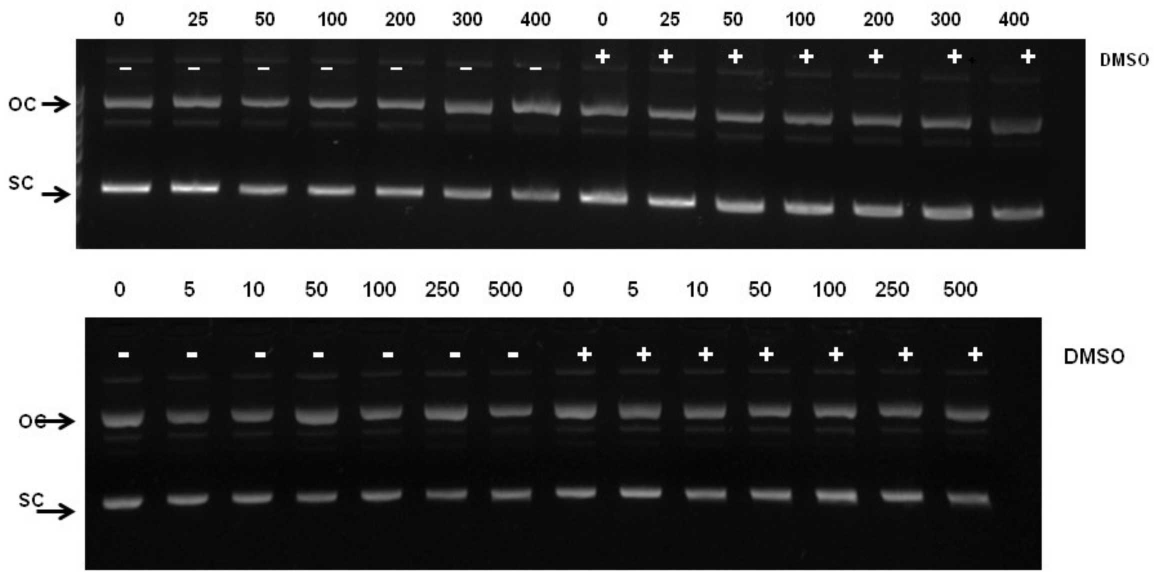

Figure S22. Cleavage of supercoiled ϕX174 DNA by $^{99m}TcO_4^-$. Incubation was performed for 24h with different activities (in μCi), at 4°C in Tris. HCl buffer (pH 7.4), in the presence or absence of DMSO (0.2M). SC and OC are supercoiled and open circular forms of DNA, respectively.

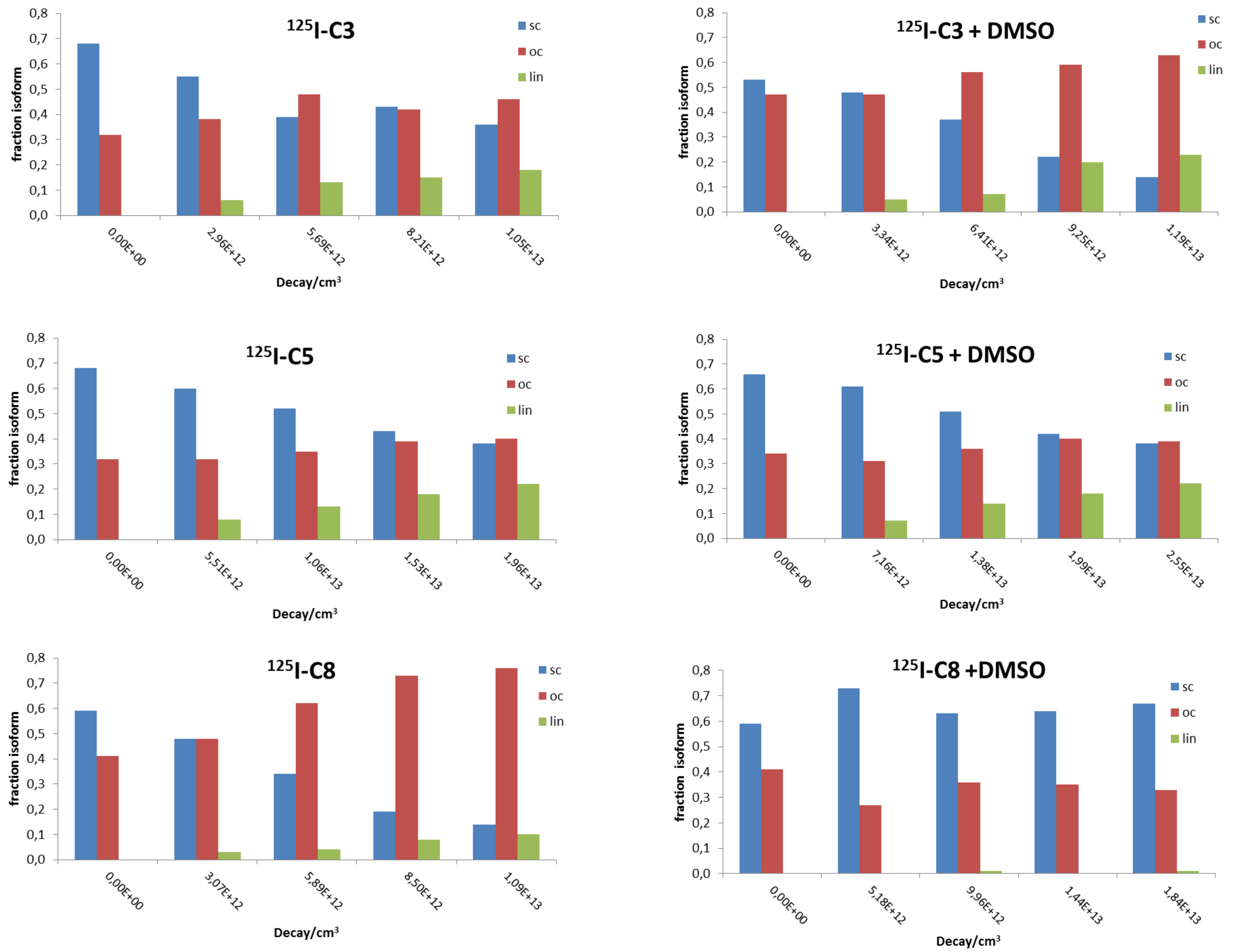

Figure S23. **Quantification of DNA isoforms resulting from the incubation of supercoiled ϕX174 DNA with ^{125}I-C$_3$, ^{125}I-C$_5$ and ^{125}I-C$_8$. SC, OC and Lin are supercoiled, open circular and linear forms of DNA, respectively.**

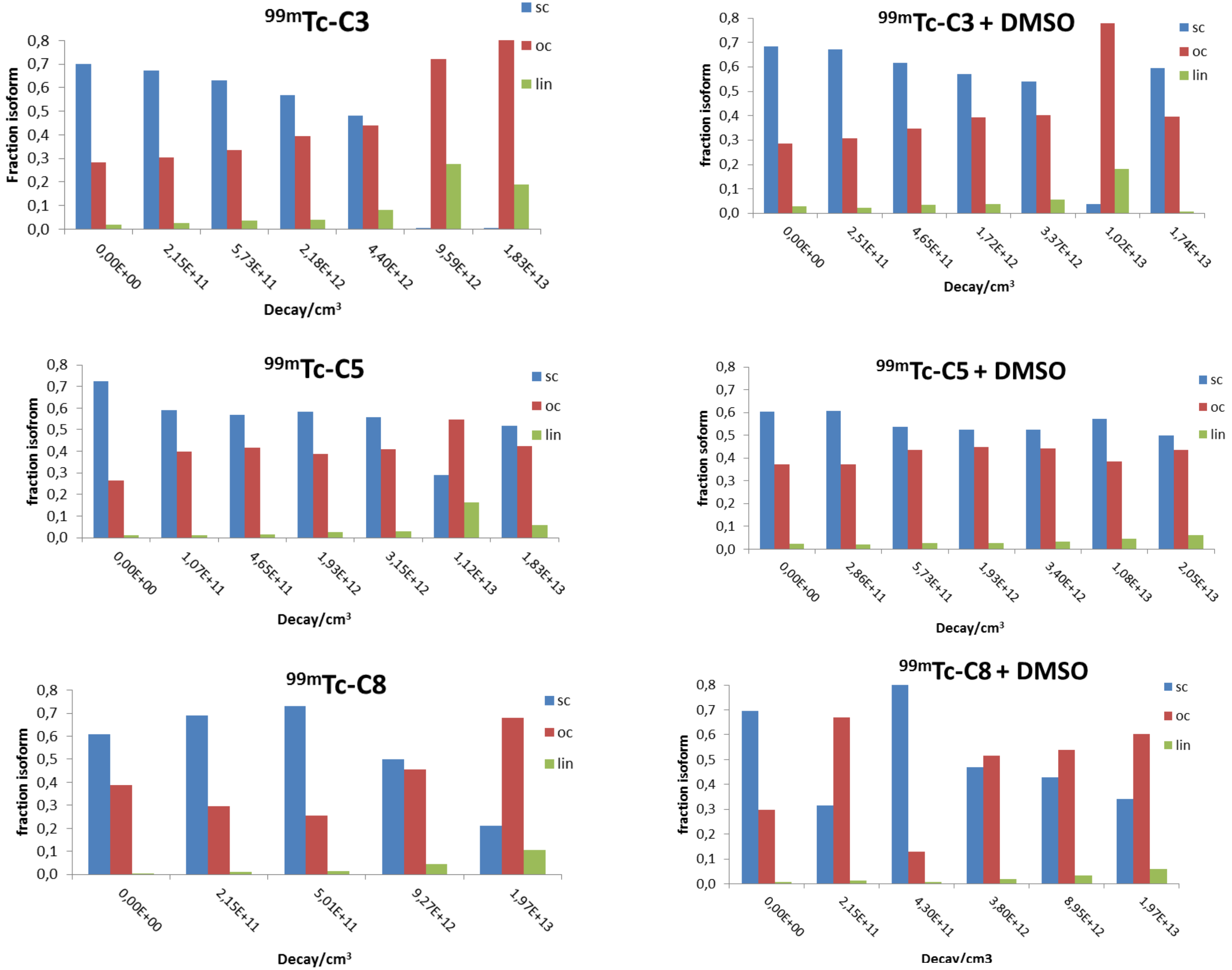

Figure S24. Quantification of DNA isoforms resulting from the incubation of supercoiled ϕX174 DNA with ^{99m}Tc-C_3, ^{99m}Tc-C_5 and ^{99m}Tc -C_8. SC, OC and Lin are supercoiled, open circular and linear forms of DNA, respectively.

a) b)

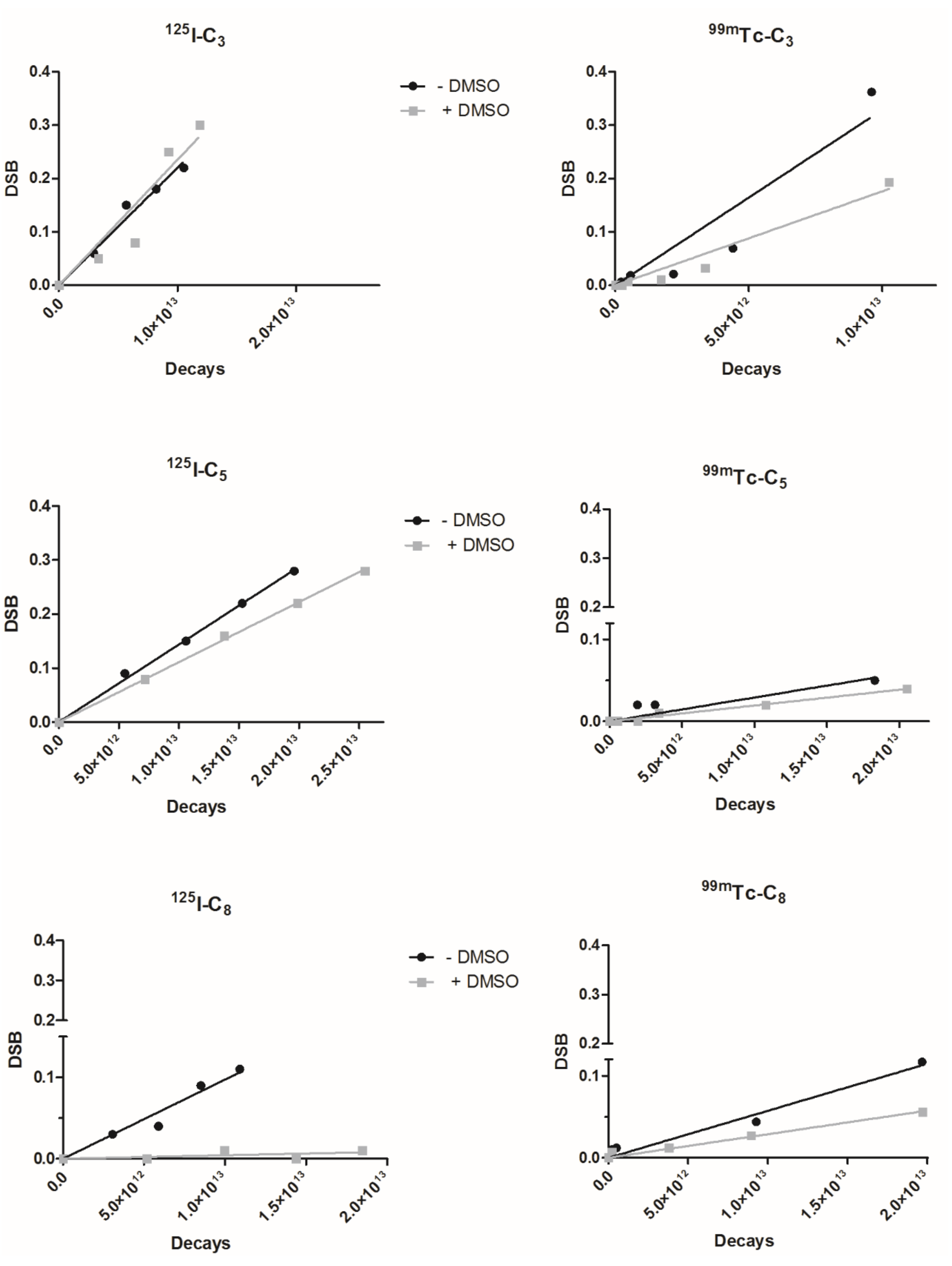

Figure S25. Number of DSBs *versus* the number of accumulated decays. ϕX174 DNA was incubated with the different radiolabelled AO derivatives, in the presence or absence of DMSO at 4°C in Tris.HCl buffer (pH 7.4): (a) ^{125}I derivatives; (b) ^{99m}Tc(I) complexes.

5. Monte Carlo (MC) Simulation: geometrical setup for MC simulations

Deposited energies were calculated in a volume corresponding to the DNA segment of 10 base pairs of length and a nucleosome, both modelled as liquid water cylinders with nanometric dimensions [16]. Liquid water is the main constituent of the human body and represents a good approximation for soft biological tissue [17].

Figure S26 illustrates the geometrical setup of the MC simulations performed with the MCNP6 code. The DNA segment was modelled as a water cylinder of 2.3 nm of diameter and 3.4 nm of height. This cylinder was set inside another water cylinder with 6 nm of diameter and 10 nm of height, which is equivalent to the size of a nucleosome. The axes of the DNA segment and nucleosome were aligned along the z-axis.

The Auger electron source was simulated as an isotropic source at different distances to the DNA axis, both for the ^{125}I and ^{99m}Tc Auger, Coster-Kronig and super Coster-Kronig energy spectra [18]. For each MC simulation 105 particles were simulated. This value was chosen being a compromise between the computational time and the statistical uncertainties obtained (between about 1% and 3%).

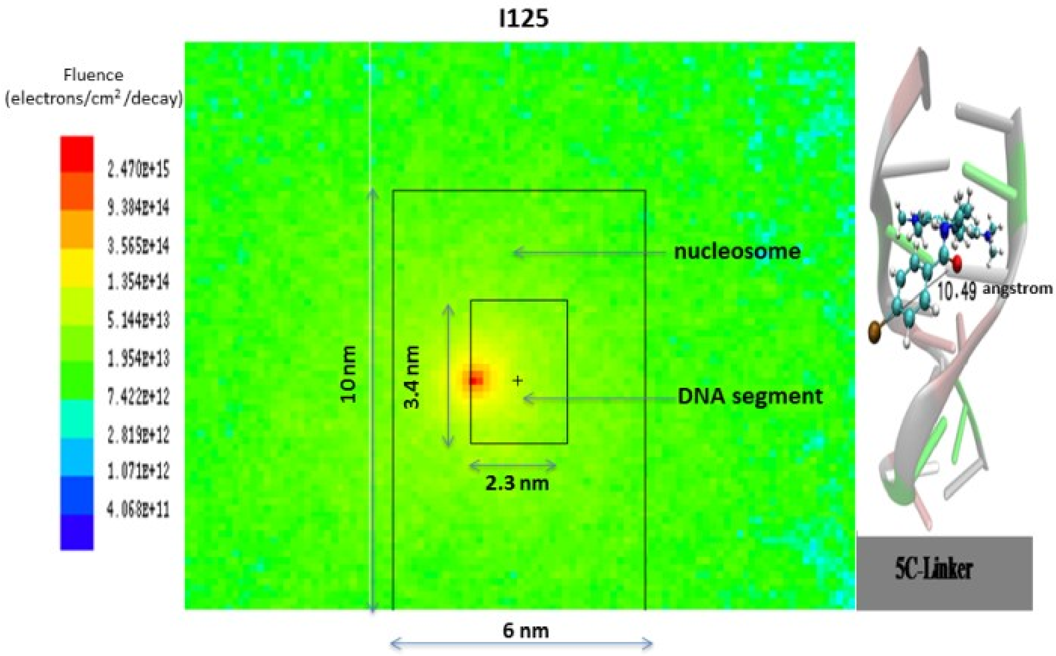

Figure S26. Geometry setup used for MCNP6 MC simulations. The ^{125}I isotropic source in this example configuration is placed at 10.49 Angstrom from the DNA segment axis. Mesh tally values around the decay source represent the intensity of electron fluence.

6. Cell studies

Assessment of DNA damage by γ-H2AX assay

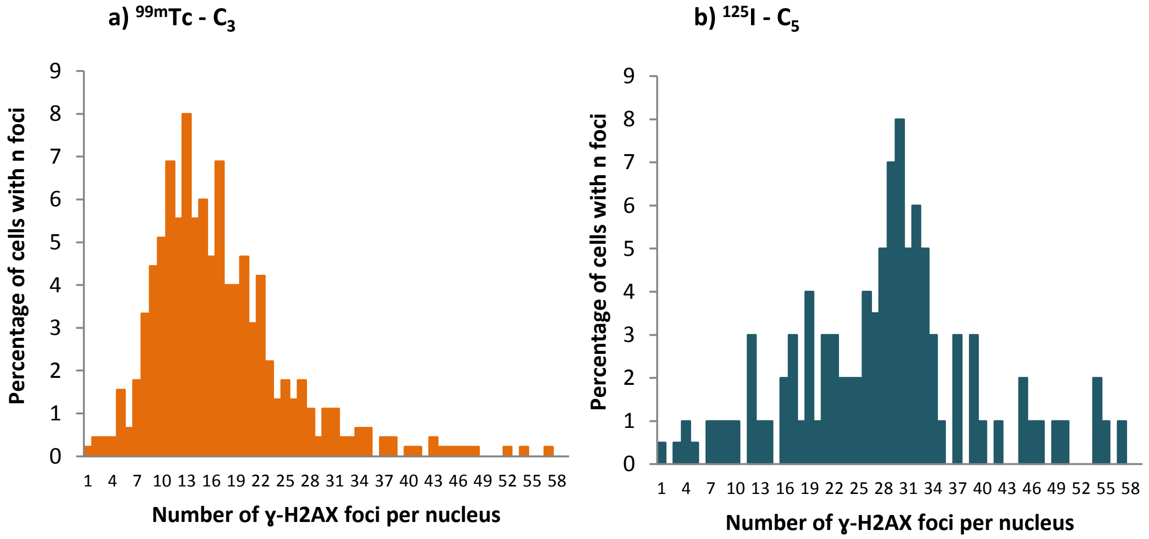

Figure S27. Quantification of DNA damage by ^{99m}Tc-C$_3$ and ^{125}I-C$_5$ in PC3 cells. Graphics show the percentage of cells with n foci after 24h of incubation with: b) ^{99m}Tc-C$_3$; b) ^{125}I-C$_5$.

7. References

1. Moura, C. *et al.* Synthesis, characterization and biological evaluation of tricarbonyl M(I) (M = Re, Tc-99m) complexes functionalized with melanin-binding pharmacophores. *New J Chem* **34**, 2564-2578 (2010).

2. Lazarova, N., James, S., Babich, J. & Zubieta, J. A convenient synthesis, chemical characterization and reactivity of [Re(CO)(3)(H2O)(3)]Br: the crystal and molecular structure of [Re(CO)(3)(CH3CN)(2)Br]. *Inorg Chem Commun* **7**, 1023-1026 (2004).

3. Gama, S. *et al.* Synthesis and biological studies of pyrazolyl-diamine Pt(II) complexes containing polyaromatic DNA-binding groups. *Chembiochem* **13**, 2352-2362 (2012).

4. Lockett, M. R., Phillips, M. F., Jarecki, J. L., Peelen, D. & Smith, L. M. A tetrafluorophenyl activated ester self-assembled monolayer for the immobilization of amine-modified oligonucleotides. *Langmuir* **24**, 69-75 (2008).

5. Eckelman, W. C., Volkert, W. A. & Bonardi M., True radiotracers: are we approaching theoretical specific activity with Tc-99m and I-123? *Nucl Med Biol* **35**, 523–527, (2008).

6. Coutinho, A. & Prieto, M. Ribonuclease T1 and alcohol dehydrogenase fluorescence quenching by acrylamide: A laboratory experiment for undergraduate students. *J Chem Edu* **70**, 425 (1993).

7. Valeur, B. & Berberan-Santos, M. N. 592 (Wiley, VHC, 2012).

8. Marquês, J. T. & de Almeida, R. F. M. Application of Ratiometric Measurements and Microplate Fluorimetry to Protein Denaturation: An Experiment for Analytical and Biochemistry Students. *J Chem Edu* **90**, 1522-1527 (2013).

9. Santos, N. C., Prieto, M. & Castanho, M. A. R. B. Quantifying molecular partition into model systems of biomembranes: an emphasis on optical spectroscopic methods. *Bba-Biomembranes* **1612**, 123-135 (2003).

10. McGhee, J. D. & von Hippel, P. H. Theoretical aspects of DNA-protein interactions: co-operative and non-co-operative binding of large ligands to a one-dimensional homogeneous lattice. *J Mol Biol* **86**, 469-489 (1974).

11. Garbett, N. C., Ragazzon, P. A. & Chaires, J. B. Circular dichroism to determine binding mode and affinity of ligand-DNA interactions. *Nat Protoc* **2**, 3166-3172 (2007).

12. Costa Pessoa, J., Correia, I., Goncalves, G. & Tomaz, A. I. Circular Dichroism in coordination compounds *J Argent Chem Soc* **97**, 151-165 (2009).

13. Robinson, H., Priebe, W., Chaires, J. B. & Wang, A. H. Binding of two novel bisdaunorubicins to DNA studied by NMR spectroscopy. *Biochem* **36**, 8663-867 (1997).

14. Balagurumoorthy, P., Chen, K., Bash, R. C., Adelstein, S. J. & Kassis, A. I. Mechanisms underlying production of double-strand breaks in plasmid DNA after decay of 125I-Hoechst. *Radiat Res* **166**, 333-344 (2006).

15. Balagurumoorthy, P., Xu, X., Wang, K., Adelstein, S. J. & Kassis, A. I. Effect of distance between decaying (125)I and DNA on Auger-electron induced double-strand break yield. *Int J Radiat Biol* **88**, 998-1008 (2012).

16. Lazarakis, P. *et al.* Comparison of nanodosimetric parameters of track structure calculated by the Monte Carlo codes Geant4-DNA and PTra. *Phys Med Biol* **57**, 1231 (2012).

17. Dingfelder, M. *et al.* Comparisons of calculations with PARTRAC and NOREC: transport of electrons in liquid water. *Radiat Res* **169**, 584-594 (2008).

18. Kereiakes, J. G. & Rao, D. V. Auger electron dosimetry: Report of AAPM Nuclear Medicine Committee Task Group No. 6. *Med Phy* **19**, 1359-1359 (1992).
